# Coupling of sea level and tidal range changes, with implications for future water levels

**DOI:** 10.1038/s41598-017-17056-z

**Published:** 2017-12-05

**Authors:** Adam T. Devlin, David A. Jay, Stefan A. Talke, Edward D. Zaron, Jiayi Pan, Hui Lin

**Affiliations:** 1 The Chinese University of Hong Kong, Shatin, New Territories, Hong Kong, SAR China; 2 Portland State University, Portland, OR United States of America; 3 College of Marine Science, Nanjing University of Information Science and Technology, Nanjing, 210044 China; 4 Shenzhen Research Institute, The Chinese University of Hong Kong, Shenzhen, Guangdong, 518057 China

## Abstract

Are perturbations to ocean tides correlated with changing sea-level and climate, and how will this affect high water levels? Here, we survey 152 tide gauges in the Pacific Ocean and South China Sea and statistically evaluate how the sum of the four largest tidal constituents, a proxy for the highest astronomical tide (HAT), changes over seasonal and interannual time scales. We find that the variability in HAT is significantly correlated with sea-level variability; approximately 35% of stations exhibit a greater than ±50 mm tidal change per meter sea-level fluctuation. Focusing on a subset of three stations with long records, probability density function (PDF) analyses of the 95% percentile exceedance of total sea level (TSL) show long-term changes of this high-water metric. At Hong Kong, the increase in tides significantly amplifies the risk caused by sea-level rise. Regions of tidal decrease and/or amplification highlight the non-linear response to sea-level variations, with the potential to amplify or mitigate against the increased flood risk caused by sea-level rise. Overall, our analysis suggests that in many regions, local flood level determinations should consider the joint effects of non-stationary tides and mean sea level (MSL) at multiple time scales.

## Introduction

Worldwide, ocean tides have exhibited trends over the past century that cannot be explained by the orbital mechanics underlying predictable tidal variability^1,2^. Regional studies have shown changes in major diurnal and semidiurnal tides in the Eastern Pacific^3^, in the Gulf of Maine^4^, in the North Atlantic^5,6^, in China^7,8^, in Japan^9^, and at islands throughout the Pacific^10^. Alongside these changes, mean sea level (MSL) has been increasing at a global rate of +1.7 ± 0.2 mm yr^−1^ as estimated from coastal and island tide gauge measurements from 1900–2009, and +3.4 ± 0.4 mm yr^−1^ for 1993–2016 as estimated from satellite altimetry^11,12^. A recent calculation shows the satellite era rate to be closer to +3.0 mmyr^−1^, though the last decade showed a faster rise, presumably from increased ice melt in Greenland^13^. However, this increase is not spatially uniform; Western Pacific MSL rise rates exceed +10 mmyr^−1^ in some locations, whereas Eastern Pacific rates are often zero or negative^14,15^. Climate models predict that MSL rates will accelerate in upcoming decades through global climate change mechanisms^16^ such as ice sheet melt and thermosteric MSL rise due to upper-ocean warming^17–24^.

Both MSL and tides exhibit short-term (seasonal to decadal) variability, in addition to long-term trends^25^. Tide properties can be altered by depth changes^26^ including those caused by MSL rise^27^, or by mechanisms driving MSL rise, such as increased surface water temperatures^28^. In either case, societal consequences are often due to total sea level, which is the sum of MSL and the time-variable water level (including tides). Therefore, both tides and MSL should be considered together in assessing the significance of future sea-level changes^29^.

**Figure 1:**
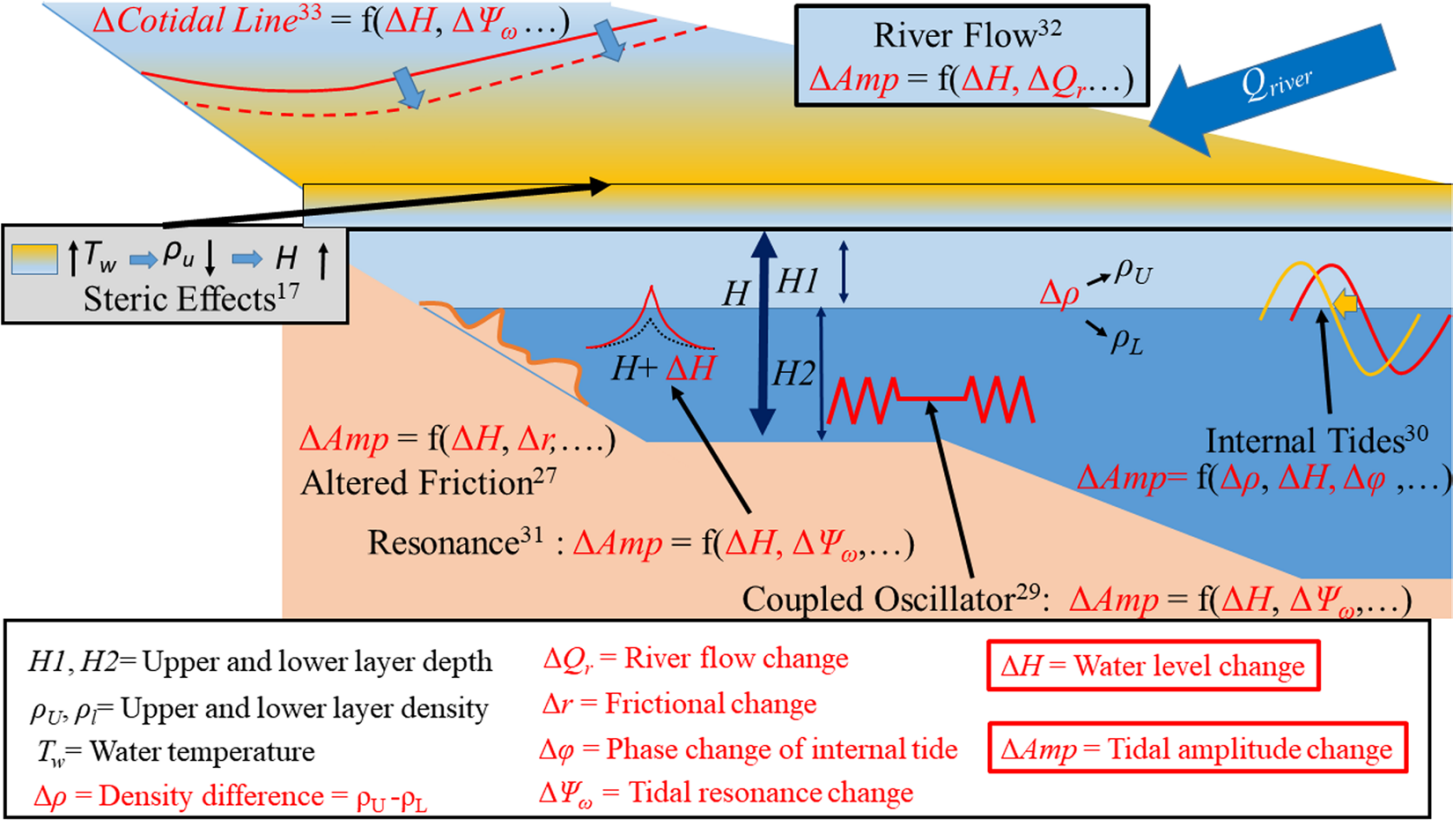
Schematic cartoon showing some of the mechanisms that can affect MSL and tides. See text above for details. Relevant reference citation numbers are indicated in figure by superscript.

Figure 1 shows a simple cartoon of the various mechanisms that can affect MSL and tides. Changes in water depth (Δ*H*) can be due to changes in density (Δ*ρ*), which is a function of water temperature (*T*_*w*_). An increase in *T*_*w*_ will decrease the density of the upper layer (*ρ*_*u*_), increasing Δ*H* ^17^. Altered frictional effects (Δ*r*) can manifest from changes in Δ*H* ^27^. The “coupled oscillator” effect between the shelf regions and the open ocean can lead to modifications of tidal amplitudes via changing Δ*r* and frequency-dependent tidal responses to astronomical forcing (Δ*Ψ*_*ω*_) for multiple tides^29^. Internal tides can change via modulations of Δ*H* and Δ*ρ*, which can change the wave phase relative to the barotropic tide, which can modulate the surface expression of the tidal amplitude^30^. Resonance is a function of Δ*H* and Δ*Ψ*_*ω*_; small changes in Δ*H* may change the resonance of enclosed bays and harbors through modification of Δ*Ψ*_*ω*_ that may either increase or decrease tidal amplitudes^31^. Water depth changes are also dependent on Δ*Q*_*r*_ in estuarine locations. An increase in Δ*Q*_*r*_ will certainly increase MSL locally, but the increased friction of the incoming tide interacting with the outgoing river discharge will change Δ*r*, decreasing tidal amplitude^32^. Finally, on a larger scale, changes in Δ*H* can also affect the structure of basin-wide amphidromic systems via movement of the co-tidal lines^33^. Another mechanism not shown on this figure is that of resonant triad interactions, such as between the M_2_, K_1_, and O_1_ tides, which can be amplified through changes in ΔH and Δ*ρ* that change local geometry and modulate all three tidal amplitudes in the relationship^25^. The correlations of multiple forcing mechanisms make it challenging to determine the causes behind the linked variability of MSL and tides that is observed at many locations^34,35^. However, the presence of correlations between sea-level and tides indicates that one or more of these processes is at work.

In summary, previous studies suggest that changes in the amplitude and phase of a tidal constituent may be a function of multiple variables:

1 $${{\rm{\Delta }}\mathrm{Amp}}_{tidal}=f({\rm{\Delta }}H,{\rm{\Delta }}{Q}_{r},{\rm{\Delta }}\rho ,{\rm{\Delta }}{m}_{x},{\rm{\Delta }}r,{{\rm{\Delta }}{\rm{\Psi }}}_{\omega },\mathrm{...})$$

where *H* is the water depth, *ρ* is water density, *r* is friction, *m*_*x*_ is mixing, *Q*_*r*_ is river discharge, and *Ψ*_*ω*_ represents frequency-dependent tidal response to astronomical tidal forcing. The “…” indicates other variables not listed here, such as wind. Since many of the variables which affect sea-level (river flow, density, wind) can also affect tidal amplitudes, a correlated response is frequently observed.

**Figure 2:**
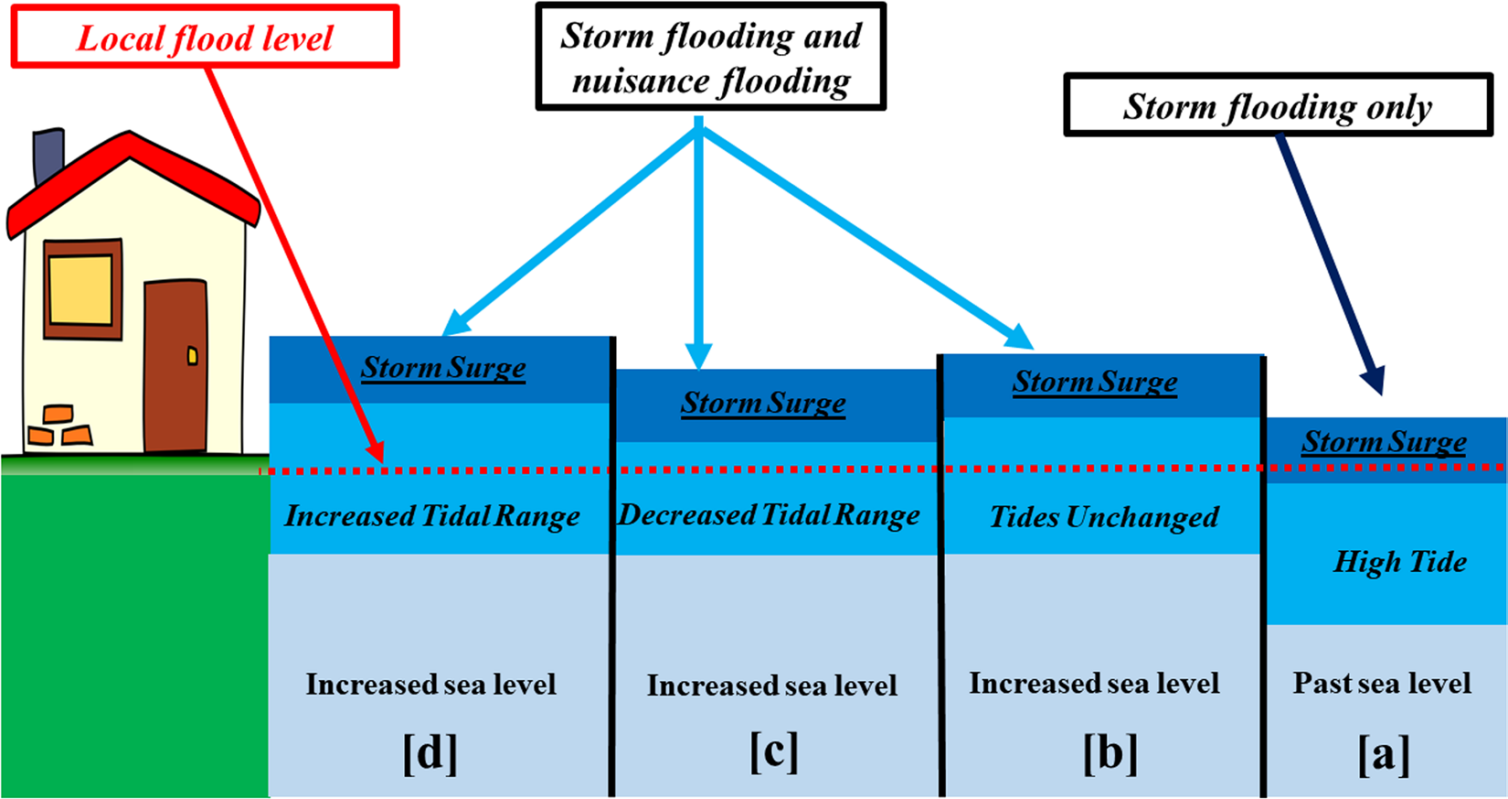
Conceptual view of the effect of nuisance flooding under four situations. In the past, when sea levels were lower, it would take a large storm surge, such as a hurricane or typhoon to cause nuisance flooding as in (**a**), but as sea levels have risen, nuisance flooding may happen at high tide as in (**b**). If tidal range becomes damped as MSL rises, then situation (**c**) arises, where nuisance flooding is still present, but not as much as in (**b**). If tidal amplitudes also increase and MSL increases, then flooding will be additionally extreme as in (**d**). The red dashed line indicates the local flood level, which was only exceeded by storm surge in the past (as in (**a**)), but under modern MSL conditions is exceeded to varying degrees in situations (**b**) through (**d**).

Identifying connections and correlations between tidal range and MSL is critical for making reliable predictions of coastal water levels and inundation risk. When combined with storm surge, larger tides and higher MSL may amplify flood risk, coastal inundation, damage to infrastructure, and population displacement. Even without the consideration of storm surge, changes in tides and sea-levels may lead to more occurrences of nuisance flooding, also known as “sunny day flooding”, which now occurs at high tide many locations, particularly along the US East and Gulf coasts^36–38^. Numerous factors contribute to nuisance flooding, including ocean swell and wave setup, local wind forcing, river flow, and the degradation of natural and man-made barriers. Hence, the joint effects of changes in tidal range and MSL are significant and of widespread relevance. Figure 2 shows four conceptually possible relationships of MSL rise and tidal change.

## Coupled changes of tides and MSL

Tidal range changes are quantified using tidal anomaly correlations (TACs), the ratio of short-term variability of tidal admittances to short-term MSL variability after removing long-term trends^25,34,35^. This method determines the sensitivity of tidal constituents to sea-level perturbations. Determinations of MSL and tidal variability are computed from harmonic analyses of hourly water level at 152 Pacific Ocean tide gauge stations, using the absolute value of the complex amplitude sum of the four largest tidal components (the two largest semidiurnal constituents, M_2_ and S_2_, the two largest diurnal components, K_1_ and O_1_), and yearly MSL. The M_2_, S_2_, K_1_, and O_1_ variabilities are summed (as complex amplitudes) to produce a combined tidal variability that is compared to MSL, and is used here as an approximation for the change in the highest astronomical tide per unit sea-level rise (δ-HAT). The combination of these four tides is approximately 75% of the sum of amplitudes determined from all constituents, and is a relatively stable time series. Theoretically, the four constituents will not be exactly in phase more than once during every 18.6-year nodal cycle, though the constituents may be approximately aligned more often; therefore, this summation provides a suitable proxy for the envelope of possible tidal amplitudes. Details of the tidal admittance, TAC and δ-HAT methods used are provided in the Methods section and in the supplementary material.

Here, we assume that the interannual variability captured by the δ-HATs can be extrapolated to the longer time scales, subject to the qualification that the changes remain “small-amplitude”. This is a reasonable assumption for many processes (e.g., reduction in frictional damping caused by higher sea-level, or the resonance response to small depth changes), but this may not be appropriate in other cases, for example, when tidal anomalies are caused by the changed phasing of internal waves^31^. To give a scale to the small-amplitude assumption, we suggest that a 0.5 to 1 m change in MSL and a change in tidal range of a few 100 s of mm is small amplitude for our purposes. Thus, we report δ-HATs in units of mm m^−1^. It will be shown later in this manuscript that this assumption is supported by the observations at Hong Kong and Honolulu. While the observed variability in tidal amplitudes over the period of record is always less than ±0.3 m for all stations, the above units are chosen for convenience, and we do not claim to be able to accurately extrapolate outside the observed range of variability.

Further, the analyses are standardized by only considering the last 30 years of a tidal record. For gauges with a length of record less than 30 years, all data are used in the δ-HATs determinations, though no gauge record less than 20 years length is used to avoid bias by the 18.61-year nodal cycle. This approach facilitates comparisons between stations and regions, and focusses attention on the part of the records most relevant to future conditions. A standardized sampling window also minimizes variations caused by other factors—such as timing errors (that are now generally smaller than in earlier periods)^10^, unrecognized gauge movement, and anthropogenic changes in the environment that can cause changes to tides. Details of the steps taken in our analyses are provided in the Methods section below, and the metadata for each station are provided in Table S1 in the supplementary material.

At three long-record gauges (San Francisco, Honolulu, and Hong Kong), the effect on total sea level (TSL) is examined by using the full tidal spectrum. All major diurnal tidal constituents (D_1_), semidiurnal constituents (D_2_), shallow-water overtides (OT), and MSL are considered separately, then all tidal components and MSL are combined to determine the TSL variability. A probability density function is calculated for MSL, D_1_, D_2_, OT, and TSL to find exceedance levels for all years of data, as well as for subsets of 20 years of data stepped by 10 years, showing historical changes in exceedance levels. The details of these calculations are provided in the Methods section.

## Results

### Pacific Ocean δ-HAT results

**Figure 3:**
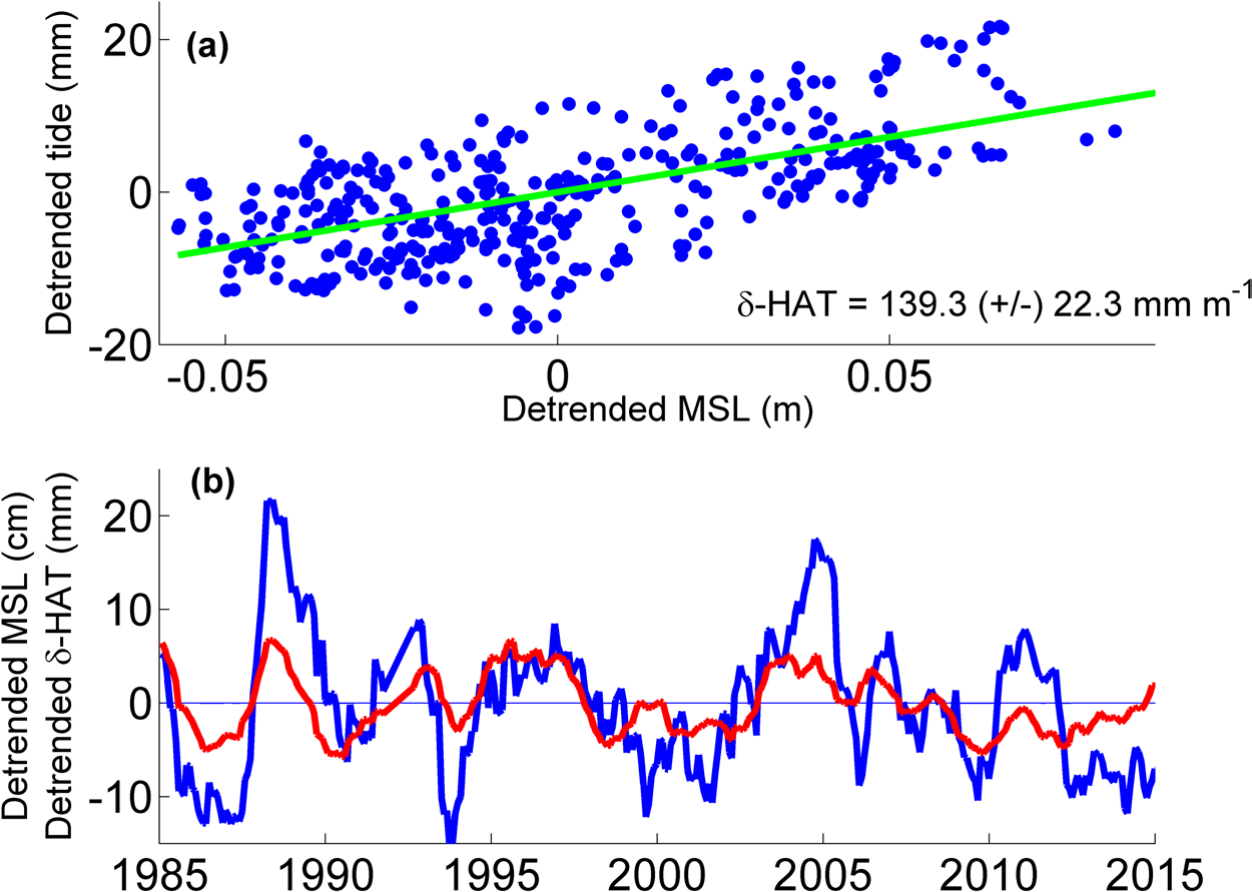
**V**ariability of δ-HAT at Honolulu, HI. In (**a**), the M_2_, S_2_, K_1_, and O_1_ amplitude variability are combined into a single detrended dataset (in units of millimeters) and regressed against the detrended mean sea level (in units of meters) to determine δ-HAT and error (units of mmm^−1^). In (**b**), the same detrended combined tidal amplitude variability is plotted in blue (units of mm), and the detrended MSL is plotted in red (units of cm) to show the time-series view. Plots were generated using MATLAB version R2011a (www.mathworks.com).

**Figure 4:**
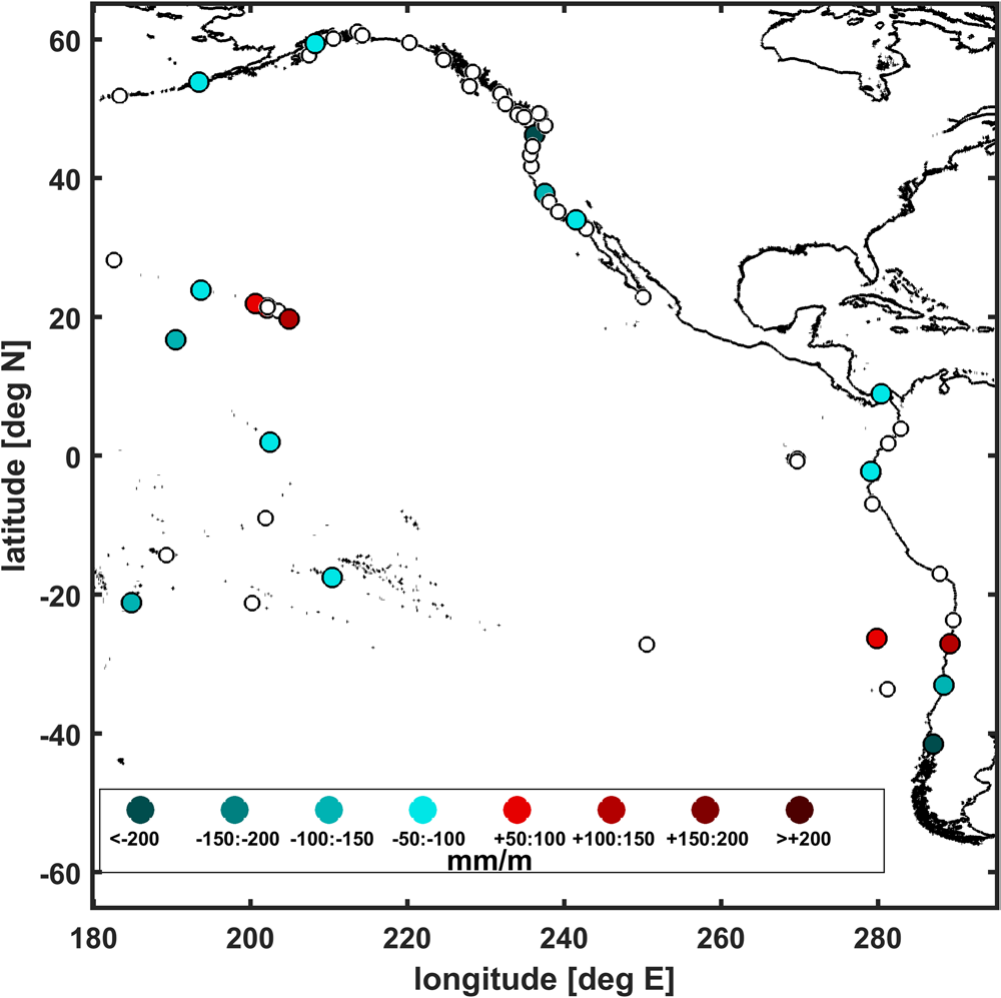
Color scale map of the Eastern Pacific δ-HAT determinations (in mm m^−1^), based on the combined M_2_, S_2_, K_1_, and O_1_ detrended tidal variations. Red and blue colored markers show positive and negative δ-HATs, respectively. Un-colored, open circles indicate that the calculated δ-HATs was not significant (*p* > 0.05); see text for details. Maps were generated using MATLAB version R2011a (www.mathworks.com).

**Figure 5:**
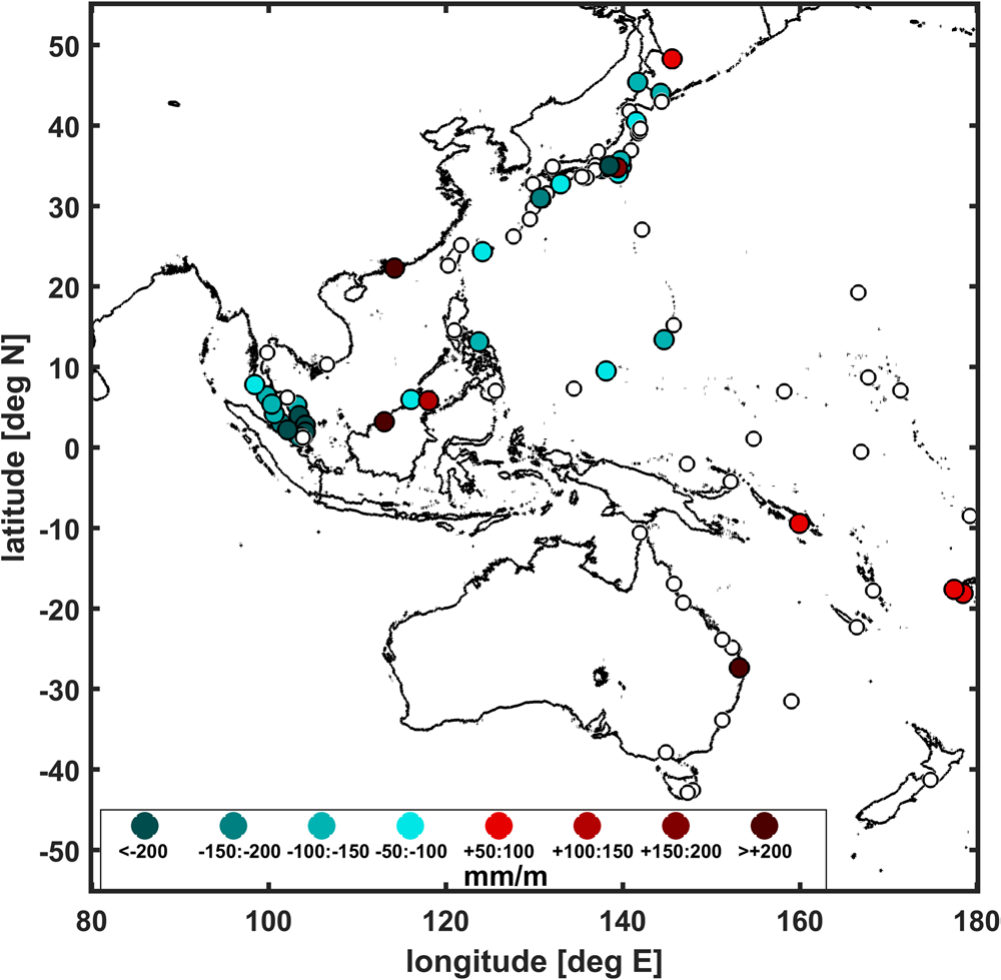
Color scale map of the Western Pacific δ-HAT determinations (in mm m^−1^), symbols and colors as in Fig. 4. Maps were generated using MATLAB version R2011a (www.mathworks.com).

We perform our yearly analyses at one-month time steps, but we only consider one determination per year in the calculation of the correlations and errors (30 total data points) to minimize any effects of autocorrelation in overlapping analysis windows. We only consider correlations that are significant as determined by a *p*-level of <0.05. Furthermore, the definition of the year used for harmonic analysis may have an influence on the value of the δ-HAT, i.e. calendar year (Jan-Dec) vs. water year (Oct-Sep), therefore, we take a set of determinations of the correlations using twelve distinct year definitions (i.e., one year windows of Jan-Dec, Feb-Jan, …, Dec-Jan.), and take the average of this set of determinations as the magnitude of the δ-HAT, and the interquartile range of the set as the confidence interval of the δ-HAT, which gives an estimate of the systematic errors present at each gauge. Details of this procedure are given in the Methods section. We illustrate an example of a δ-HAT calculation at Honolulu, HI (Fig. 3). A scatter plot shows the positive correlation (Fig. 3a) between detrended sea-level (cm) and detrended tidal range (mm), and a linear regression shows a slope of +139.5 ± 21.6 mm per meter of sea-level anomaly; this is the δ-HAT. The time series of the tide anomaly generally follows the sea-level anomaly; when sea level has a positive anomaly, tidal amplitudes are elevated, and vice-versa (Fig. 3b). The full-sampled monthly-stepped results are shown in the scatterplot, but the overlaid trend (green line) is based on the sub-sampled data set described above. A similar analysis is performed at all 152 stations. Ocean-wide, the results of the δ-HAT calculations in the Eastern Pacific (Fig. 4) and Western Pacific (Fig. 5) reveal that tides at 54 stations (~35%) are significantly correlated to variations in sea-level, with δ-HATs >±50 mm m^−1^ (i.e., >±5% of the sea level perturbation). The exact δ-HAT values, their confidence intervals and exact *p*-values are listed in Table S2 in the supplementary material.

In the Eastern Pacific (Fig. 4), significant δ-HATs are scattered and isolated. San Francisco (CA) and Astoria (OR), exhibit δ-HAT values of −146 ± 35 and −257 ± 35 mm m^−1^, respectively. Honolulu and Hilo, HI have positive values of +139 ± 22 and +147 ± 12 mm m^−1^, respectively. Along the coast of South America, there is an anomalously large negative anomaly correlation at far-southern Puerto Montt, CHL, with a δ-HAT value of −963 ± 108 mm m^−1^ and a lesser negative trend is also seen at Valparaiso, CHL, though there are positive δ-HATs of +80 ± 36 and +127 ± 27 mm m^−1^ at San Felix Island and Caldera, CHL further north. Elsewhere in the Eastern Pacific of note, Johnston Island and Papeete (Tahiti), PYF exhibit large negative δ-HATs of −117 ± 24 mm m^−1^ and −95 ± 43 mm m^−1^, respectively.

The δ-HAT correlations are more numerous in the Western Pacific (Fig. 5). Eleven gauges in Japan show negative δ-HATs, seven of which are greater than −100 mm m^−1^, with a maximum negative value of −351 ± 29 mm m^−1^ occurring at Maisaka. Only two significant positive δ-HATs are observed in Japan, at Okada and Hanasaki (+159 ± 20 and +54 ± 17 mm m^−1^). At Western Pacific islands, results are mixed, with moderate positive δ-HATs found at Honiara in the Solomon Islands (+67 ± 10 mm m^−1^) and Suva in Fiji (+72 ± 68 mm m^−1^), and moderate negative δ-HATs observed at Guam (−116 ± 17 mm m^−1^), Christmas Island (−71 ± 8 mm m^−1^),and Yap Island (−58 ± 25 mm m^−1^). Within the South China Sea, the distribution of δ-HATs is complex. An anomalously large positive δ-HATs is observed at Hong Kong (+665 ± 99 mm m^−1^) and at Bintulu, MYS (+615 ± 141 mm m^−1^), and a lesser positive value occurs at Sandakan, MYS (+108 ± 28 mm m^−1^). Both sides of the Malay peninsula exhibit strongly negative δ-HATs. The Malacca Strait on the west side of the peninsula has δ-HATs of ~−70 to −220 mm m^−1−^,and the Gulf of Thailand on the eastern side sees δ-HATs of ~−130 to −290 mm m^−1^; both sides increase gradually from the northern reaches to the southern tip of the Malay Peninsula. Elsewhere in the Southwest Pacific, there is a significant positive δ-HAT at Brisbane, AUS (+272 ± 40 mm m^−1^), and a significant negative δ-HAT at Legaspi (−145 ± 36 mm m^−1^), PHL.

### Impacts of changes in tides on total sea levels (TSL)

The impact of tide/sea-level correlations on total sea level (TSL) exceedance is investigated using three long-record gauges with significant δ-HATs: Honolulu (+139 ± 22 mm m^−1^; ~110 years), San Francisco (−147 ± 35 mm m^−1^, ~120 years), and Hong Kong (+665 ± 99 mm m^−1^, ~50 years). A full tidal analysis is employed at these stations, considering all tidal constituents and MSL, and then considering the sum of MSL and all tides (TSL). The 90%, 95%, and 99% exceedance levels for each water level component (D_1_, D_2_, OT and MSL) were calculated in 20-year segments, starting with the beginning of the record, and stepping forward 10 years at a time. Because results are similar, we only report the 95% exceedance level here, and list the other levels in the supplementary material. Comparisons are then made between the earliest 20-year segment and the most recent 20-year segment to determine the long-term change of the exceedance levels.

At all three gauges, MSL shows a steady increase at the 95% exceedance level, reflecting the sustained rise in global sea levels. The exceedance levels of tides, however, show a mixture of increases and decreases. At Honolulu, where δ-HAT is moderately positive, the increase in the MSL 95% exceedance level since 1905 is +112 mm. For the tidal components, the D_1_ exceedance level has stayed nearly constant (+2 mm), the D_2_ level has shown a moderate increase (+17 mm), and the OT level has decreased slightly (−3 mm). Summing up all tidal components and MSL gives a change in the TSL level at Honolulu of +124 mm. Compared to the MSL level changes, the TSL exceedance change is greater by +12 mm, a relative increase of +11% as compared to MSL change alone. At San Francisco, where δ-HAT is moderately negative, the MSL exceedance change since 1897 is +188 mm. For the tides, the D_1_ exceedance level has decreased (−10 mm), the D_2_ level has increased (+30 mm), and the OT level has decreased (−27 mm). After consideration of all water level components, the TSL level at San Francisco has increased by +194 mm. When compared to MSL, a small difference in the level changes of +6 mm is seen, indicating a small relative increase of +3% as compared to MSL alone. Finally, Hong Kong, where δ-HAT is strongly positive, exhibits very strong changes in exceedance levels, and all water level components are positive since 1965. The change in exceedance levels are: MSL, +78 mm; D_1_, +36 mm; D_2_, +22 mm; and OT, +51 mm. The TSL exceedance level has increased by +150 mm, a relative change of +92% as compared to MSL rise alone. Table 1 lists the 95% exceedance level changes for MSL vs. TSL at Hong Kong, Honolulu and San Francisco. Full tables showing all changes (D_1_, D_2_, OT, MSL, TSL) in the 90%, 95%, and 99% exceedance levels are included in the supplementary material (Tables S3, S4 and S5).

**Table 1:** Historical 95% exceedance level changes of MSL vs. TSL at Hong Kong, Honolulu, and San Francisco. For all determinations, the 95% level is reported for the first part of the historical tide gauge record (1965–1985 for Hong Kong; 1905–1925 for Honolulu and 1897–1917 for San Francisco), the last part of the record (1995–2015 for all three stations), and the difference between the two times to show the change in each exceedance level. All values are in units of millimeters.

	First 20-year exceedance levels (mm)	Recent 20-year exceedance level (mm)	Change in exceedance (mm)
**Hong Kong (1965–2015)**			
MSL 95% exceedance	1584	1662	+**78**
TSL 95% exceedance	3330	3480	+***150***
**Honolulu (1905–2015)**			
MSL 95% exceedance	1408	1520	+**112**
TSL 95% exceedance	1988	2098	+***124***
**San Francisco (1897–2015)**			
MSL 95% exceedance level	2708	2894	+**188**
TSL 95% exceedance level	4396	4590	+***194***

**Figure 6:**
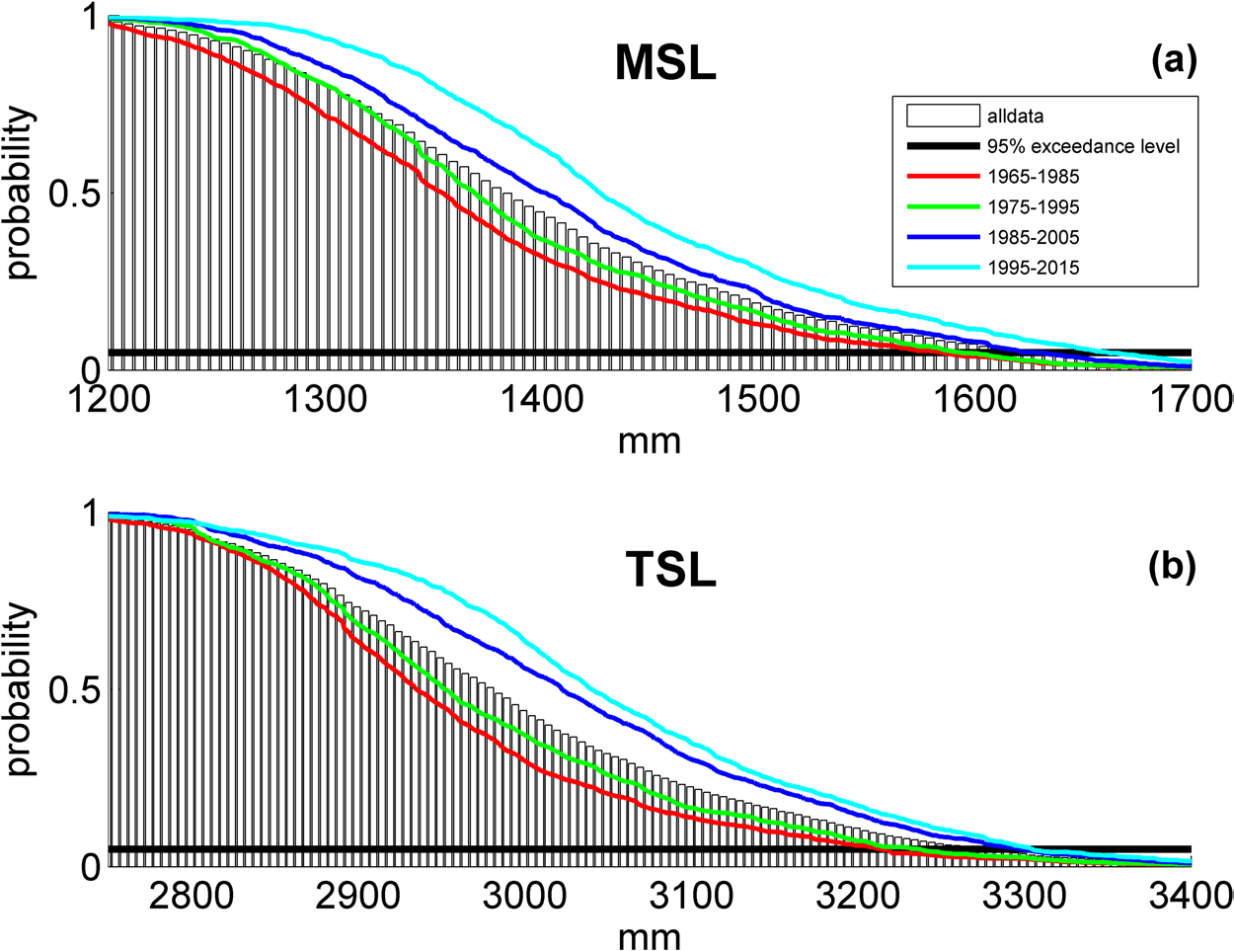
Probability density functions (PDFs) of: (**a**) mean sea level (MSL), and (**b**), total sea level (TSL) for the Hong Kong tide gauge. In both plots, the black bar graph shows the distribution of all data, red lines indicate the period of 1965–1985, green lines indicate 1975–1995, dark blue lines indicate 1985–2005, and light blue lines indicate 1995–2015. The solid horizontal black line on each subplot shows the 95% exceedance level. Plots were generated using MATLAB version R2011a (www.mathworks.com).

Figure 6 shows a visual representation of the MSL/TSL exceedance level comparisons results for Hong Kong; Fig. 6a is the MSL PDF, and Fig. 6b shows the TSL PDF. In both plots, the black bar graph shows the distribution of all data, red lines indicate 1965–1985, green lines indicate 1975–1995, dark blue lines indicate 1985–2005, and light blue lines indicate 1995–2015. It can be clearly seen that the probability distribution has increased from the early record (1965–1985, red lines) to the recent record (1995–2015, light blue line) for both MSL and TSL. The solid horizontal black line on each subplot shows the 95% exceedance level for reference. Similar plots for Honolulu and San Francisco are shown in the supplementary material (Figures S10 and S11).

We now examine how the δ-HAT determinations compare to the exceedance level changes. Honolulu, where δ-HAT is positive and moderately large (~+140 mm m^−1^) shows a positive and moderate increase in TSL exceedance as compared to MSL. Over the past century, MSL at Honolulu has risen by ~0.12 m. If this MSL change is multiplied by the δ-HAT value, it would suggest a change in tidal range of ~+18 mm, on the same order as the additional change in TSL due to tides found from the PDF determinations of exceedance levels (+12 mm; see Table S3).

At Hong Kong, where the largest positive δ-HAT in this study was found, all tidal exceedances and MSL reinforce each other, leading to large relative increases of TSL exceedances compared to MSL exceedances, which is significant considering that the record length available for Hong Kong is only about half that of Honolulu and San Francisco. In Hong Kong, between the 1965–1985 and 1995–2015 periods, local sea-level rose by ~0.15 m, with most of the change occurring in the 1990s. Multiplying δ-HAT (+665 mm m^−1^) by 0.15 m of sea-level rise yields a predicted change in tidal range of +99 mm, ~33% larger but on the same order as the exceedance level changes in TSL due to tides (+72 mm). Therefore, the observed differences in the increase of TSL, a +92% increase as compared to MSL rise alone, indicates that the overall probability of exceeding any arbitrary nuisance flooding datum has greatly increased at this location (Table 1; Supplementary Table S4).

However, at San Francisco, short-term, seasonal variations in tidal properties and tide range oppose the long-term trend (Supplementary Table S5). While tidal constituents and tidal range have increased over secular time scales^3^, wintertime floods can cause significant reductions in M_2_ up to 20%^39^. Hence, the negative δ-HAT calculated for San Francisco, if extrapolated into the future, would produce an inaccurate assessment of how sea-level rise would affect the distribution of extremes. The divergent response may reflect physical processes acting at different time scales; while wintertime river flow decreases tidal range in a predictable manner, the 30% long-term decrease in river flow at SF may slightly have increased M_2_ over longer time scales^39^. Alternately, a completely different mechanism—such as sea-level rise or channel deepening—may influence statistics over decadal time scales. Finally, prosaic details—such as movement of the SF gauge in the 1920s, or measurement quality—may slightly influence statistics over a long time scale^40^. In conclusion, the δ-HAT is more closely tied to the short-term variability here, which is dominated by river discharge, a seasonal and/or yearly variable process, and the exceedance level changes of TSL are more closely related to the significant increase of MSL over the past century and the slower long-term increases in tidal amplitudes.

## Discussion

The results of δ-HAT reveal areas of regional coherency, though no basin-scale patterns of change can be discerned. In Hawaii, the large significant positive values of δ-HATs at Honolulu and Hilo are mainly due to the M_2_ TAC, which has been related to the changing phase of the internal tide^30^. Large negative δ-HATs observed in the US are at river-influenced stations (Astoria and San Francisco), where freshwater flow increases sea-level, but river-induced frictional effects damp the tides^32,41^. There is a shortage of data along the South American Pacific coast, but some significant δ-HAT were observed along the Chilean coast. A very large negative δ-HAT is seen at Puerto Montt, CHL, in a region of decreasing sea-level, which is correlated with an increasing tidal range. The large δ-HAT value here may simply reflect the large magnitude of tides observed here (mean tide range of ~7 m), or that depth induced variability is influenced by the near-resonant geometry of the surrounding waters^42,43^. There is also a milder negative δ-HAT at Valparaiso, CHL and positive δ-HATs at Caldera and San Felix Island, though there is large geographic distance between these gauges and Puerto Montt, and it is not clear if the δ-HATs are related here to the dynamics at Puerto Montt.

A few positive δ-HATs are observed in the Coral and Solomon Sea areas of the southwest Pacific, namely, Honiara, Suva, and Brisbane. Changes in regional stratification (such as those caused by the El-Nino climate variation) were previously found to be an important factor in tidal variability in the Solomon Sea region^25^. The positive response at Brisbane may be related to water level-related changes in frictional effects in the Coral Sea due to the Great Barrier Reef, which may be inversely proportional (approximately) to mean depth via a “coupled oscillator” concept^29^. There are many large negative δ-HATs at gauges along the Pacific coast of Japan, which may be related to changes in the Kuroshio Current system^44^. Apart from Okada and Hanasaki, all significant δ-HATs on the Pacific Coast of Japan are strongly negative, suggesting a common mechanism.

On the western side of the South China Sea, gauges in Malaysia exhibit large negative δ-HATs related to the seasonal variability in tides due to stratification, seasonal monsoon winds and water depth^34^. In the eastern parts of the South China Sea, the anomalously large positive δ-HATs at Hong Kong, Sandakan, and Bintulu may be attributable to basin scale changes, but are also likely to be affected by local coastal development and land reclamation projects, particularly in Hong Kong. The regional correspondence makes a basin scale change possible; however, there are limited tide gauge data in the region to validate such a hypothesis. In other shallow, semi-enclosed regions, such as the North Sea, increasing sea-level has amplified tides on the German/Dutch coast over the past 50 years due to reduced frictional effects^45^. At the seasonal time scale, changes in stratification force alterations in the M_2_ and M_4_ tides, with peak amplitudes observed in summer^46^. On even shorter time scales, storm surge on the German coast also amplifies tides, due to temporarily increased sea-level^47^. Since the South China Sea, like the North Sea, is fringed by extensive regions of shallow water, similar mechanisms may occur and affect secular, interannual, seasonal, and even event-scale perturbations. Focused numerical modeling and more historical tide gauge data are required to test these hypotheses.

We conclude from the PDF analyses that exceedance levels of high water change over time, and that the δ-HAT statistic—based on short term anomalies—can reasonably approximate the secular shift in water level exceedance values when scaled by observed sea-level rise at some (but not all) stations. The δ-HAT, which quantifies short-term variability, can be an adequate proxy for long-term secular shifts in TSL observed using the entire tide spectrum at some locations (Hong Kong and Honolulu), whereas the short-term determinations are less reliable at long-term prediction at other locations (San Francisco) due to complex river-influenced dynamics. Nonetheless, two practical conclusions are clear: a) consideration and comparisons of δ-HAT and exceedance level changes can provide a useful measure of the sensitivity of individual locations to tidal evolution correlated with sea-level changes; and b) tidal evolution needs to be considered, along with MSL rise, in evaluating TSL, inundation risk, and associated human impacts.

Hong Kong, with a dense population and at-risk coastal infrastructure, is highly vulnerable to an amplified total sea-level (TSL) response over and above MSL rise. Probabilistic analyses of nonstationary of tides at Hong Kong shows an increase in the 95% exceedance level of TSL that is nearly double (+92%) of that determined by MSL rise alone. At this location, changes in tides are as important as the changes in MSL for assessing future sea level extremes, even without considering other parts of the water level spectrum such as surface waves (swell) and storm surge, both of which also change due to altered water levels^45^. The other locations analyzed (Honolulu and San Francisco) also exhibit century-scale changes in high water levels that, while smaller in magnitude than Hong Kong (~ 5–10%), are still significant, and would not be apparent from an assumption of stationary tides; therefore, these factors should be integrated in future coastal planning.

The δ-HATs presented in Figs 4 and 5 indicate that approximately 10% of locations analyzed are significantly vulnerable to positive feedback effects, in which higher sea-level correlates with an amplification of δ-HAT by more than 5%. Conversely, regions with negative δ-HATs (~25% of all locations), such as San Francisco and Astoria in North America, the Pacific coast of Japan, and Southeast Asia, may experience a negative feedback loop in which sea-level rise is somewhat mitigated by a decrease in tidal properties, though the example of San Francisco shows that the relationship between MSL and δ-HAT may be a function of time scale, at least when local processes river inflow are important. While more stations show a negative than a positive δ-HAT, we note this may be caused by a regional bias in station density; North American, Japanese and Malaysian stations are over-represented in the survey, while South American, Chinese and Thai stations are underrepresented, among others.

A growing concern is that climate change and sea-level rise may produce amplified TSL responses in some coastal systems, with the risk to coastal infrastructure outpacing the increased risk caused by sea-level rise alone^47^. The methods described here to predict changes in the highest astronomical tide (δ-HAT) and evaluate trends in tidal exceedance levels are new approaches to assessing total variability and time scales of variability beyond simple long-term MSL trend evaluation. Thus, this method can be applied to shorter time series to assess the risk of longer term changes, though care is needed in interpreting results, and detailed dynamical studies will be needed for this purpose in some cases. The possibility of adverse feedback effects is exemplified by the case of Hong Kong, where both the δ-HAT statistic and evaluation of a 50-year tide data set both suggest that changes to tides and sea-level both contribute to rapidly increasing exceedance levels of high water.

Careful, physically based evaluations at individual locations would be required to assess the validity of extrapolating δ-HATs to secular tide changes correlated to sea-level rise and other climate factors such as altered stratification. Furthermore, both nuisance flooding and storm surge occur over short, meteorological time scales, and a full consideration of wind, wave, and other hydrodynamic effects such as non-linear interactions are required to assess changing risk to infrastructure and human populations. Nonetheless, the results of this manuscript suggest that the non-stationarity of tides should be given greater consideration than in the past in future analyses of extreme water levels, and have suggested locations worthy of future study and numerical modelling. Century-scale changes observed in the exceedance levels at Honolulu and San Francisco are mild (~ 10%), but at Hong Kong, with an extensive coastal infrastructure, exceedance levels have nearly doubled as compared to a stationary tide. Moreover, changes in Hong Kong occurred quickly due to accelerated sea-level rise (primarily in the 1990s). Therefore, scenarios of future accelerated sea level rise should be coupled with correlated changes in tides to evaluate their impact at individual locations to guide future coastal planning.

## Methods

### Data inventory

This study examines 152 tide gauges in the Pacific Ocean. The majority of hourly tide gauge records used are from the University of Hawaii’s Sea Level Center (UHSLC). However, nearly all of the U.S. Pacific data (including San Francisco and Honolulu, where we do more detailed study of the changes in exceedance levels) is collected and quality controlled by the National Oceanographic and Atmospheric Administration (NOAA) National Ocean Service (NOS). There is also additional data from the following agencies: the Japanese Oceanographic Data Center (JODC); Canada’s Fisheries and Ocean office (FOC); and Australia’s National Tidal Center (AuNTC). The factors that guided station selection were: (1) Location: All stations used are within the Pacific Ocean and connecting minor basins. (2) Temporal coverage: All stations used have a length of record (LOR) greater than one nodal cycle (18.61 years). (3) Completeness: All stations contain more than 80% valid data over the record. Pacific gauges that meet these criteria are indicated in Fig. 4 (Eastern Pacific) and 5 (Western Pacific), and Table S1.

### Context for Equation (1)

Tidal amplitudes are a function of multiple variabilities:

2 $${{\rm{Amp}}}_{tidal}=f(H,r,{{\rm{\Psi }}}_{\omega },\mathrm{...})$$

where *H* is the water depth (including MSL, waves, storm surge, ocean stratification, river inflow, winds, etc.), *r* is friction, and *Ψ*_*ω*_ represents frequency-dependent tidal response to astronomical tidal forcing. The “…” indicates other variabilities not considered here, such as wind. To determine the possible changes in tides, the changes in these variables must be accounted for:

3 $${{\rm{\Delta }}\mathrm{Amp}}_{tidal}=f({\rm{\Delta }}H,{\rm{\Delta }}r,{{\rm{\Delta }}{\rm{\Psi }}}_{\omega },\mathrm{...})$$

However, each of these variabilities, and the changes in each, are also dependent on multiple factors, shown below:

4a $${{\rm{\Psi }}}_{\omega }=f(H,r,\mathrm{...})\to {{\rm{\Delta }}{\rm{\Psi }}}_{\omega }=f({\rm{\Delta }}H,{\rm{\Delta }}r,\mathrm{...})$$

4b $$H=f(\rho ,{Q}_{r},\mathrm{...})\to {\rm{\Delta }}H=f({\rm{\Delta }}\rho ,{\rm{\Delta }}{Q}_{r},\mathrm{...})$$

4c $$r=f(H,\rho ,\mathrm{...})\to {\rm{\Delta }}r=f({\rm{\Delta }}H,{\rm{\Delta }}\rho ,\mathrm{...})$$

The tidal response function is a function of water depth and friction, water depth is a function of buoyancy, *ρ*, and river discharge, *Q*_*r*_, and friction is a function of water depth and buoyancy. Finally, buoyancy, *ρ*, as well as the change in buoyancy, is a function of water temperature, *T*_*w*_, water salinity, *T*_*s*_, river discharge, *Q*_*r*_, and mixing, *m*_*x*_:

5 $$\rho =f({T}_{w},{S}_{w},{Q}_{r},{m}_{x},\mathrm{...})\to {\rm{\Delta }}\rho =f({\rm{\Delta }}{T}_{w},{\rm{\Delta }}{S}_{w},{\rm{\Delta }}{Q}_{r},{\rm{\Delta }}{m}_{x},\mathrm{...})$$

As with the above equations, there may be other variabilities unconsidered in this treatment. Applying the chain rule to Eq. (2), and considering the variabilities of all dependent factors will yield a general expression for the variability in tidal amplitudes, yielding our original expression for Eq. (1).

### Tidal admittance calculations

Investigations of tidal trends are carried out using a tidal admittance technique^3^. An admittance is the unitless ratio of an observed tidal constituent to the corresponding tidal constituent in the astronomical tide generating force expressed as a potential, *V*, which is divided by the acceleration due to gravity, *g*, to yield a quantity, *Z*_*pot*_(t) = *V/g*, with units of length that can be compared to tidal elevations *Z*_*obs*_(t) on a constituent by constituent basis, via harmonic analysis. Because nodal and other low-frequency astronomical variability is present with similar strength in both *Z*_*pot*_ and *Z*_*obs*_, its effects are eliminated in the yearly analyzed admittance time series, to the extent that the oceanic response to low frequency astronomical forcing is linear. The primary requirement for linearity in this context is that the hydrodynamic response to tidal forcing is independent of frequency for small changes in frequency (i.e., those related to nodal and other slow variations in tidal forcing). In monthly analyses, the use of an admittance largely eliminates the apparent seasonal variability of the analyzed constituents due to constituents not included in the analysis, e.g., the effects of P_1_ and K_2_ on K_1_ and S_2_, respectively. In our analysis, yearly tidal harmonic analyses (at monthly time steps), and monthly harmonic analyses (at weekly time steps) are performed on hourly observed tidal records and the corresponding hourly ATGF generated at the same location, using the R_T_Tide tidal harmonic analysis package in MATLAB^48,49^, along with separate code to generate the tidal potential^50,51^. The result from a single harmonic analysis of *Z*_*obs*_(t) or *Z*_*pot*_(t) determines an amplitude, *A*, and phase, *θ*, at the central time of the analysis window for each tidal constituent, along with error estimates. Analyzing the entire tide gauge record in yearly or monthly windows produces time-series of amplitude and phase. From amplitude *A*(t) and phase *θ*(t) time series, one can construct complex amplitudes *Z*(t) through:

6 $${\bf{Z}}(t)=A(t){e}^{i\theta (t)}.$$

Time-series of tidal admittance (**A**) and phase lag (**P**) for a constituent are formed using Eqs (7) and (8):

7 $${\bf{A}}(t)=abs|\frac{{Z}_{obs}(t)}{{Z}_{pot}(t)}|\,,$$

8 $${\bf{P}}(t)={\theta }_{obs}(t)-{\theta }_{pot}(t),$$

The size of the moving analysis windows chosen (yearly and monthly) are determined by the scales of variability to be examined and the distribution of astronomical tidal frequencies, and there are benefits and drawbacks to both window sizes. Yearly analyses return more tidal constituents, distinguish constituents separated by one cycle per year, resolve the nodal variability, and average out shorter-term seasonal variability, allowing a more precise analysis of decadal-scale variability. Monthly analyses return fewer constituents (i.e., capturing constituent groups separated by 1 cycle per month rather than constituents separated by one cycle per year) and are noisier, but the shorter window length captures seasonal cycles that are obscured in yearly analyses. Our approach is consistent with recommendations in the existing literature^47,48^.

The harmonic analysis that generates the **A**s and **P**s also provides an MSL time-series. For each resultant dataset (MSL, **A** and **P**), the mean and trend are removed from the time series, to allow direct comparison of their co-variability around the trend. The magnitude of the long-term trends is typically much less than the magnitude of the short-term variability, which is now more apparent in the data. The removal of a trend also reduces the effects of land motions (e.g., glacial isostatic adjustment (GIA), subsidence, and tectonic effects, assumed linear on the time scale of tidal records) that occur on longer time scales, whereas we are concerned with short-term variability.

The δ-HAT is a proxy for the change in the highest astronomical tide, which is estimated by combining the complex time series of the yearly analyzed M_2_, S_2_, K_1_, and O_1_ tides, approximately 75% of the full tidal height. “Complex” means, in this context, that each constituent is considered as a complex number (accounting for both amplitude and phase), the complex vectors are added, and the total amplitude is resolved from the complex sum. The detrended time series of δ-HAT is then compared to the detrended MSL variability. The magnitude of the slope of the regression is the definition of the δ-HAT, and we report δ-HATs in units of mm m^−1^. A detailed description of the step-by-step method, with additional figures showing the intermediate steps in the process, are provided in the supplementary materials.

Our full analyses consider one-year harmonic analyses at one-month steps, which are useful for plotting purposes. Because of probable autocorrelation between determinations due to this overlap, calculations of the regressions and associated statistics (i.e., the *p*-values) are based on a sub-sampled dataset of one determination per year, for 30 data points in a 30-year record. However, the definition of the year window used for harmonic analysis may have an influence on the value of the δ-HAT, i.e. calendar year (Jan-Dec) vs. water year (Oct-Sep). To provide a better estimate of the overall correlations for all data we take a set of determinations of the correlations using twelve distinct year definitions (i.e., 30 one-year windows running from Jan-Dec, Feb-Jan, …, Dec-Jan.). We then take the average of this set as the magnitude of the δ-HAT. For an estimate of the confidence interval, the interquartile range (middle 50% of the set range) as the confidence interval of the δ-HAT. We only consider correlations to be significant if they have a *p*-level of <0.05, as well as having a magnitude of greater than ±50 mm m^−1^.

### Full Tidal Analyses and PDFs

The PDFs of tide distributions are produced using all tidal constituents with a signal-to-noise ratio greater than 2.0. Monthly tidal harmonic analyses reveal both seasonal and inter-annual tidal variability (see above in this section), but trade time resolution for accuracy; as a result, many lesser-amplitude constituents are poorly resolved. Therefore, to obtain a fully resolved PDF, we infer the amplitude of lesser constituents using harmonic analysis based on a year of data, and assume they are constant over the year. Monthly analyses are used for the six largest tidal components (M_2_, S_2_, N_2_, K_1_, O_1_, and Q_1_) and ten overtide components (M_4_, M_6_, M_8_, MK_3_, MO_3_, SK_3_, MN_4_, MS_4_, SN_4_, and S_4_). The primary constituents typically have a strong signal (i.e., a signal-to-noise ratio ~100) even for only a month of data, and even the majority of overtides used here will have signal-to-noise ratios of at least 2.0. However, lesser constituents might not be significantly resolved in monthly analyses. Therefore, we supplement this data by using the results of the yearly harmonic analyses for 20 additional diurnal tides (α_1_, 2Q_1_, NO_1_, J_1_, OO_1_, υ_1_, σ_1_ ρ_1_, τ_1_, β_1_, χ_1_, π_1_, P_1_, ψ_1_, φ_1_, θ_1_, and SO_1_), 14 semidiurnal tides (L_2_, T_2_, μ_2_, η_2_, OQ_2_, ε_2_, 2N_2_, ν_2_, H_1_, H_2_, MKS_2_, λ_2_, R_2_, and K_2_), and 2 additional overtides (MK_4_, SK_4_), which are then interpolated to the monthly time scale. The total diurnal (D_1_) variability is then summed over all constituents into a single time-series, as are the semidiurnal (D_2_) and overtide (OT) components. Finally, all tidal and MSL variability is summed into a single time series to yield total sea level (TSL). For each independent harmonic analysis, any constituents which do not return a valid value (either zero magnitude or signal-to-noise ratio <2) are not included in the final calculations. In our consideration of extreme water levels, the value of the 90%, 95%, and 99% exceedance levels are calculated (though only the 95% level is discussed since results are similar for all three calculations), and the changes in the MSL exceedance are compared to the TSL exceedances to show the importance of non-stationary tides.

## Electronic supplementary material

Supplementary Materials
