# Supplementary material for: Coupling of sea level and tidal range changes, with implications for future water levels
Source: Sci Rep. 2017 Dec 5;7:17021. doi: 10.1038/s41598-017-17056-z (PMC5717176; doi:10.1038/s41598-017-17056-z)
Supplement: Supplementary file 1 — Supplementary Materials [file 41598_2017_17056_MOESM1_ESM.pdf]

Coupling of sea level and tidal range changes with implications for future water levels

Adam T. Devlin[1, 2, *] David A. Jay[2], Stefan A. Talke[2,] Edward D. Zaron[2], Jiayi Pan[1], Hui Lin[1]

[1] - The Chinese University of Hong Kong, Shatin, New Territories, Hong Kong, SAR

[2] - Portland State University, Portland, OR, United States of America

[*] - Corresponding author

SUPPLEMENTARY MATERIAL

S1. Methodology Supplement

S1.1 Tidal admittance calculations

Investigations of tidal trends are carried out using a tidal admittance. An admittance is the unitless ratio of an observed tidal constituent to its corresponding astronomical tide generating force (ATGF) expressed as a tidal potential, *V/g*. Because nodal and other low-frequency astronomical variability is present with similar strength in both the observed tidal record and in *V/g*, it is largely absent in admittance time series. The variations that remain are some combination of noise and non-astronomical signals, which allows useful examination of longer term climate trends.

Harmonic Analysis techniques (HA) rely upon having a long enough record to be able to accurately separate relevant frequencies of interest. An inherent trade-off exists between obtaining multiple estimates (good for assessing variability) and accuracy. For analyses of record lengths much longer than the frequency of interest, the harmonic constants are well-determined and highly accurate, but the number of determinations is low. In analyses of shorter record length, frequencies can be "contaminated" by the "satellites" (constituents that have nearly the same frequency), but can yield more determinations and data points.

For all relevant stations in the Pacific, overlapping yearly tidal harmonic analyses (at monthly time steps), and monthly harmonic analyses (at weekly time steps) were carried out on hourly observed tidal records and the corresponding hourly ATGF generated at the same location, using the R_T_Tide tidal harmonic analysis package in MATLAB (Pawlowicz,2002; Leffler and Jay 2009). *V* is determined based on the methods of Cartwright and Tayler (1971), and Cartwright and Edden (1973) using software obtained from Dr. Richard Ray of NASA (personal communication with David Jay, 2007). The result from a single harmonic analysis determines an amplitude, A, and phase, θ, at the central time of the analysis window for each tidal constituent, with error estimates. Use of a moving analysis window produces time-series of amplitude and phase for both annual and monthly analyses. An example is shown for the amplitude of the K_1 constituent at San Francisco in Figure S3, shown for both the gravitational potential (top), and the observed data (bottom).

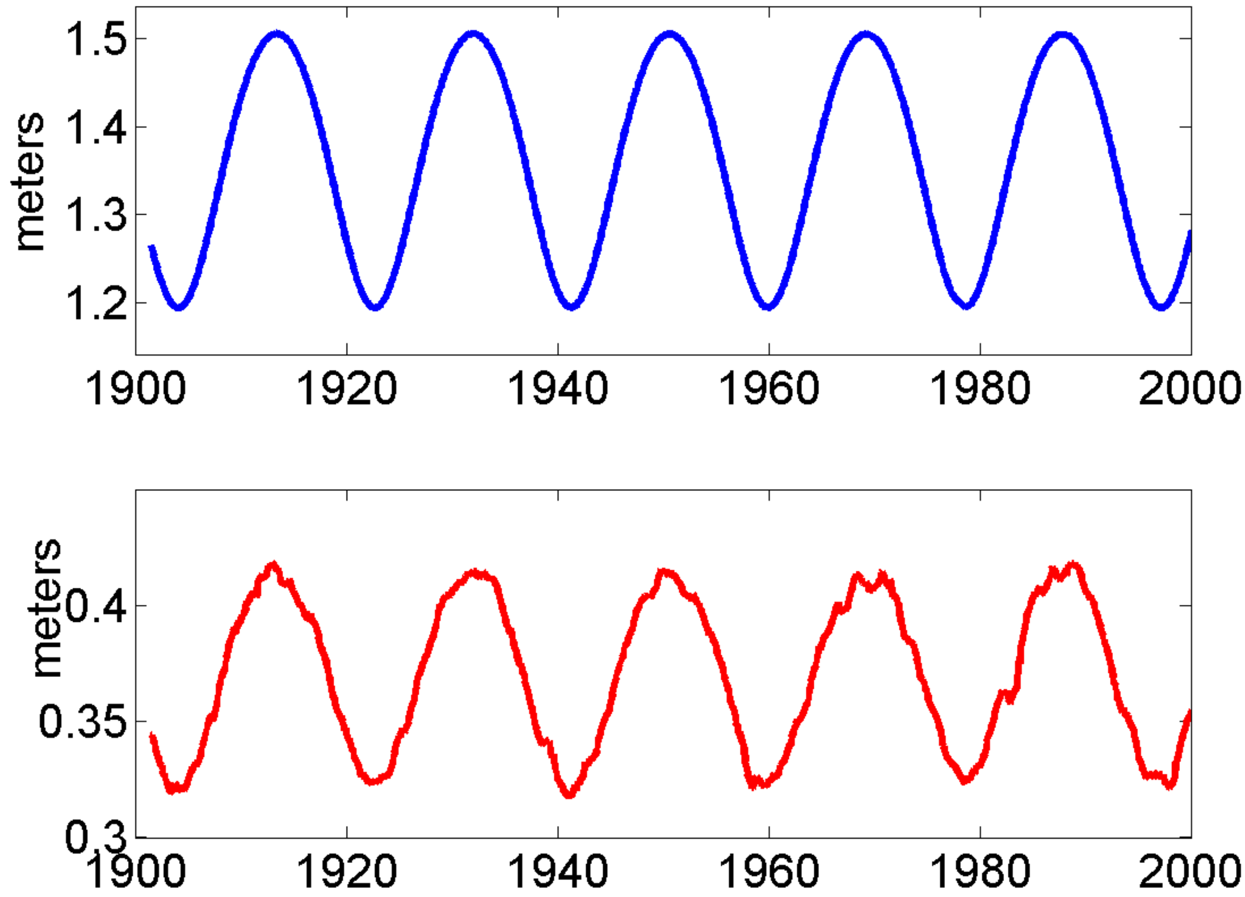

Figure S3 Amplitude of the K_1 constituent at San Francisco, CA generated from tidal harmonic analysis, for the gravitational potential (top), and observed hourly tidal data (bottom).

From amplitude *A*(t) and phase *θ*(t) time series, one can construct complex amplitudes *Z*(t) through:

$$\mathbf{Z}(t) = A(t)e^{i\theta(t)} \tag{1}$$

Time-series of tidal admittance ratio (**A**) and phase difference (**P**) for a constituent are then formed using Eqs. (2) and (3):

$$\mathbf{A}(t) = abs \left| \frac{Z_{obs}(t)}{Z_{pot}(t)} \right| \tag{2}$$

$$\mathbf{P}(t) = \theta_{obs}(t) - \theta_{pot}(t) \tag{3}$$

where the subscript 'obs' or 'pot' denotes the corresponding quantity for either the observed data or the gravitational potential, respectively. Both the admittance ratio in Eq. (2) and the phase difference in Eq. (3) largely remove the nodal-cycle variability in yearly analyses, and the yearly variability in monthly analyses, allowing easier examination of the non-astronomic signals. The mean value of the tidal potential at each location is used as a scale factor to convert the **A** into a "true" amplitude. The K_1 **A** at San Francisco generated from the potential and observed data shown in Figure S3 is presented in Figure S4, with a linear trend of +0.033 mmyr^{-1} overlain in green.

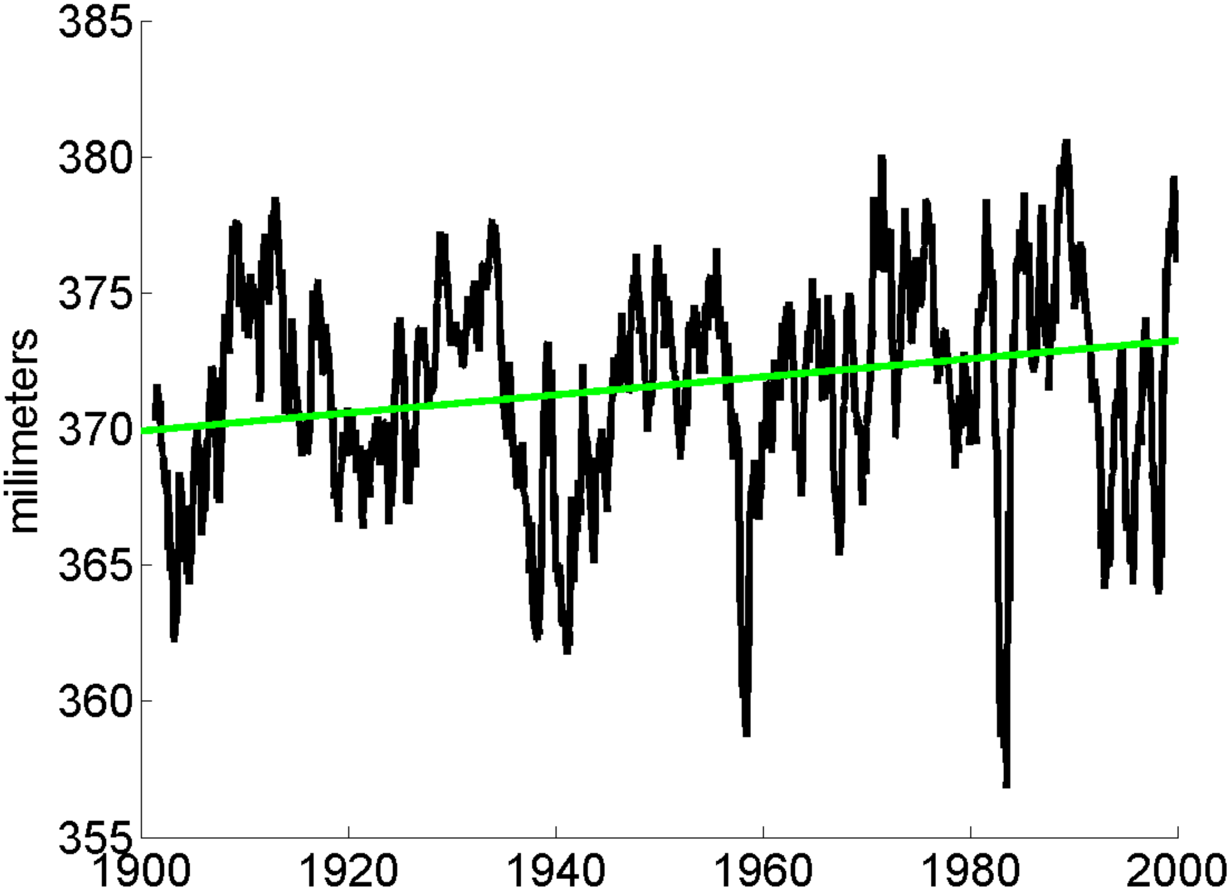

Figure S4 Admittance amplitude ratio (**A**) of the K_1 constituent at San Francisco, CA, calculated from Eq. (2), and the potential and observed data shown in Figure S3, with a linear trend of +0.033 mmyr^{-1} overlain in green.

Once the **A**s and **P**s are generated, MSL time-series are also generated, through either yearly or monthly averaging of hourly water level records, shown for San Francisco in Figure

S5, with a linear trend of +1.8 mmyr^{-1} overlain in red. For each dataset, a mean and a long-term trend are estimated over the desired length of the tide record (here, we show the entire SF record, but in the manuscript, the TACs or δ-HATs are calculated only over the past 30 years of the record). The mean and trend are then removed from the time series, to allow direct comparison of their variability. The magnitude of the long-term trends is typically much less than the magnitude of the short-term variability, the latter of which will now be more apparent in the data. The removal of trends also removes the effects of land motion that may contaminate MSL records. Detrended quantities for our example at San Francisco are shown in Figure S6 (K_1 **A**), and Figure S7 (MSL).

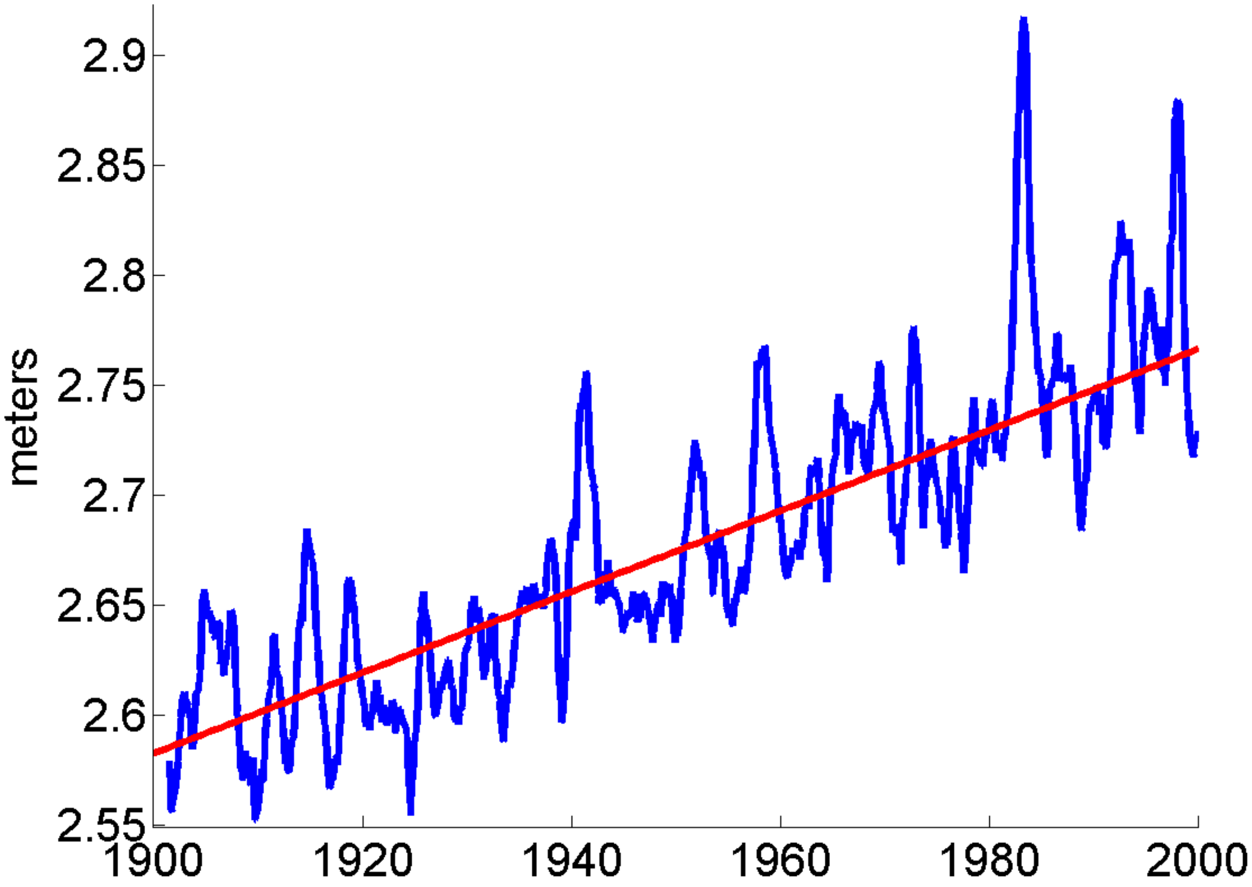

Figure S5 Mean sea level (MSL) at San Francisco, CA, yearly averaged, with a linear trend of +1.8 mmyr^{-1} overlain in red.

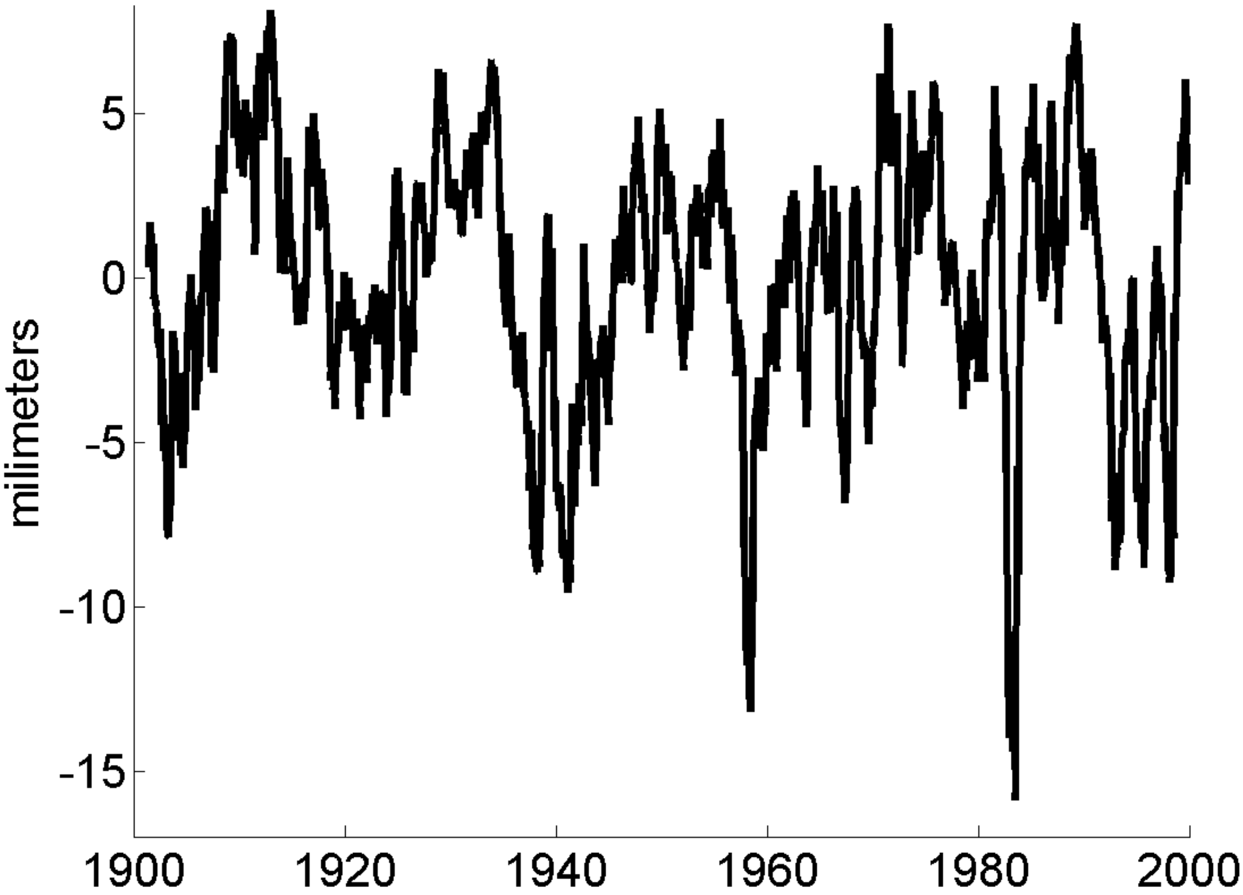

Figure S6 Detrended admittance amplitude ratio (**A**) of the K_1 constituent at San Francisco, CA.

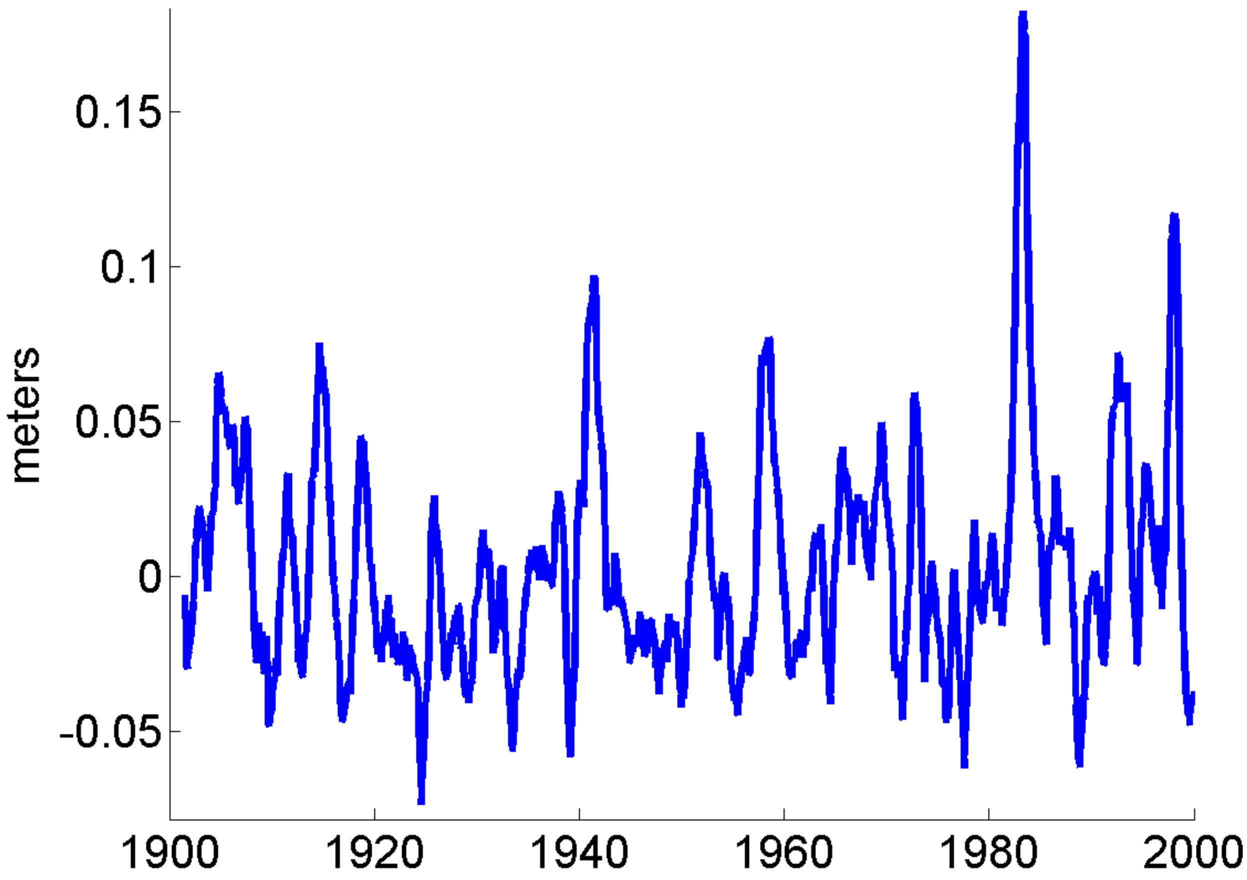

Figure S7 Detrended mean sea level (MSL) at San Francisco, CA.

S1.2 Annual scale correlation of tidal variations and MSL

Our primary interest is the interannual variations and not the long-term trends. The detrended time-series of residual variations in **A** and **P**, and residual variations in MSL, can now be examined for coherence with one another (and with other ancillary time-series), using scatter plots, cross-correlations, and regression statistics generated from trend determination. Figure S8 shows the detrended K_1 **A**, and the detrended MSL at San Francisco plotted on the same axis, with the MSL anomalies in units of cm, and the tidal amplitude anomalies in units of mm. Figure S9 shows a scatterplot between both detrended quantities at San Francisco (**A**; mm vs. MSL; m). We define the tidal anomaly correlation (TAC) as the slope between detrended tidal properties and detrended MSL, here, for tidal amplitudes, expressed as the millimeter change in tidal amplitude per meter of sea level rise (mmm^{-1}). The same approach could be used with the phase difference time-series to provide phase anomaly trends, with the trends expressed as degree

change in tidal phase per meter of sea level rise (degm^{-1}). The errors of the TAC determinations are defined as the 95% confidence interval (CI) of the linear trend determination. Trends are deemed significant if the signal-to-noise ratio (SNR) of the linear trend to the associated error is greater than 2.0.

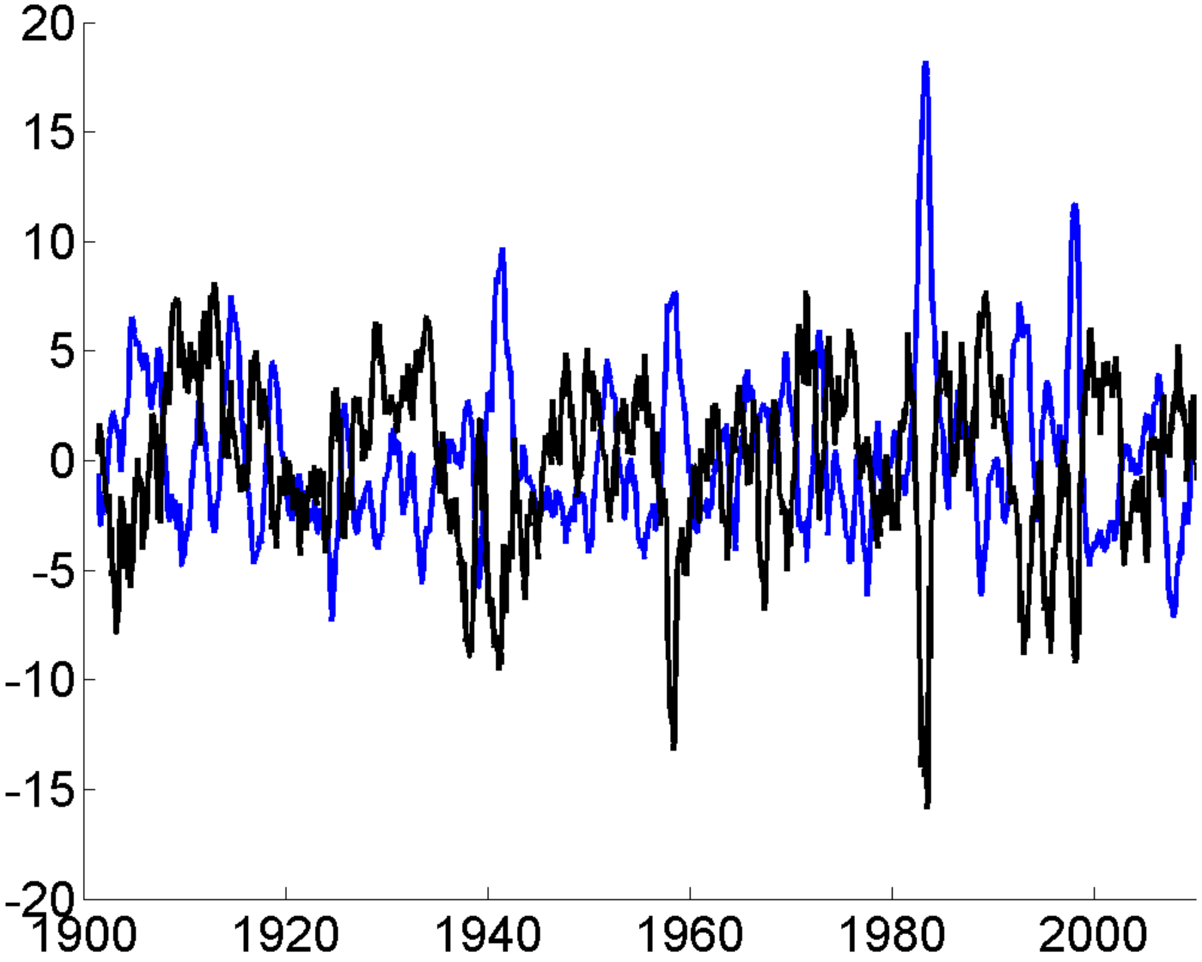

Figure S8 Detrended MSL (blue, units of cm) plotted with detrended K_1 **A** (black, units of mm) at San Francisco, CA.

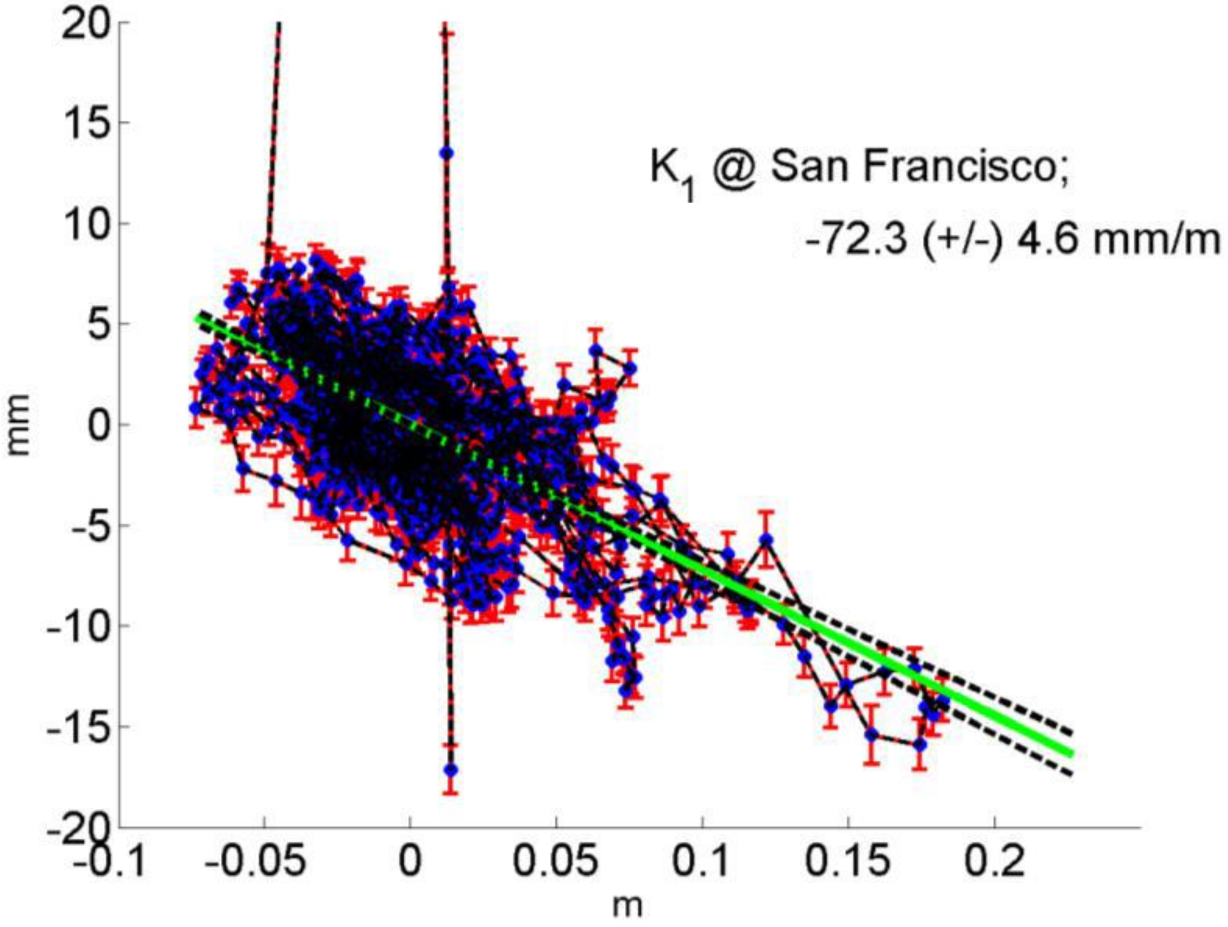

Figure S9 Example TAC of detrended MSL (meters) scattered against detrended K_1 **A** (millimeters) at San Francisco, CA, with a value of -72.3 ± 4.6 mmm^{-1}.

For our example of the K_1 **A** vs. MSL at San Francisco, it is clearly seen that the trend is negative, i.e., times of positive MSL anomaly correspond to occurrences of negative K_1 **A** anomaly. The TAC, determined from the slope, is: -72.3 ± 4.6 mmm^{-1}. Finally, to produce the δ-HATs used in this study, we perform the above steps on each tidal constituent, but combine the M_2, S_2, K_1, and O_1 variabilities (e.g., Figure S6) into a single time series before detrending and comparing to the detrended MSL variability. All other steps of the methodology will then be applied to produce the δ-HAT, with results like that shown in Figure S9.

Our full analyses consider one-year harmonic analyses at one-month steps, which are useful for plotting purposes. Because of probable autocorrelation between determinations due to this overlap, calculations of the regressions and associated statistics (i.e., the *p*-values) are based on a sub-sampled dataset of one determination per year, for 30 data points in a 30-year record.

However, the definition of the year window used for harmonic analysis may have an influence on the value of the δ-HAT, i.e. calendar year (Jan-Dec) vs. water year (Oct-Sep). To provide a better estimate of the overall correlations for all data we take a set of determinations of the correlations using twelve distinct year definitions (i.e., 30 one-year windows running from Jan-Dec, Feb-Jan, …, Dec-Jan.). We then take the average of this set as the magnitude of the δ-HAT. For an estimate of the confidence interval, the interquartile range (middle 50% of the set range) as the confidence interval of the δ-HAT. We only consider correlations to be significant if they have a p-level of < .05, as well as having a magnitude of greater than ±50 mm m^{-1}.

S1.3 Probability density function (PDF) analysis of water level exceedance

For the probability density function analyses, we consider the complete tidal variability. Individual variabilities are combined to produce a time series using all resolvable constituents for both the yearly and monthly analyses. The time-series of the six largest constituents in the monthly analyses (M_2, S_2, N_2, K_1, O_1, and Q_1) as well as ten overtide components (M_4, M_6, M_8, MK_3, MO_3, SK_3, MN_4, MS_4, SN_4, and S_4) are first combined into a diurnal (D1; K_1, O_1, and Q_1), semidiurnal (M_2, S_2, and N_2), and overtide (OT) band. We supplement this data by using the results of the yearly harmonic analyses for 20 additional diurnal tides (α_1, $2Q_1$, NO_1, J_1, OO_1, υ_1, σ_1 ρ_1, τ_1, β_1, χ_1, π_1, P_1, ψ_1, φ_1, θ_1, and SO_1), 14 semidiurnal tides (L_2, T_2, μ_2, η_2, OQ_2, ε_2, $2N_2$, ν_2, H_1, H_2, MKS_2, λ_2, R_2, and K_2), and 2 additional overtides (MK_4, SK_4), which are then interpolated to the monthly time scale of the D_1, D_2, and OT time-series. Finally, monthly averaged MSL determinations are used in the generation of the MSL time series.

The hybrid time series of tidal variability and MSL are then used to develop a probability density function (PDF) calculation of all tides and MSL (total sea level; TSL), which shows the relative likelihood that a value will be above or below a certain value. In our consideration of extreme water levels, the value of the 90%, 95%, and 99% exceedance levels are calculated, and the changes in the MSL exceedance are compared to the TSL exceedances to show the importance of considering non-stationary tides. In the main text, we report only the changes in the 95% exceedance levels, and highlight the difference between the MSL and TSL exceedance level changes over the historical record using Table 1, and give a closer look at Hong Kong using Figure 6. Here, we also show the PDF comparisons of MSL and TSL at Honolulu and San

Francisco (Figures S10 and S11), with exact values of all components of exceedance level change reported in Tables S3, S4, and S5.

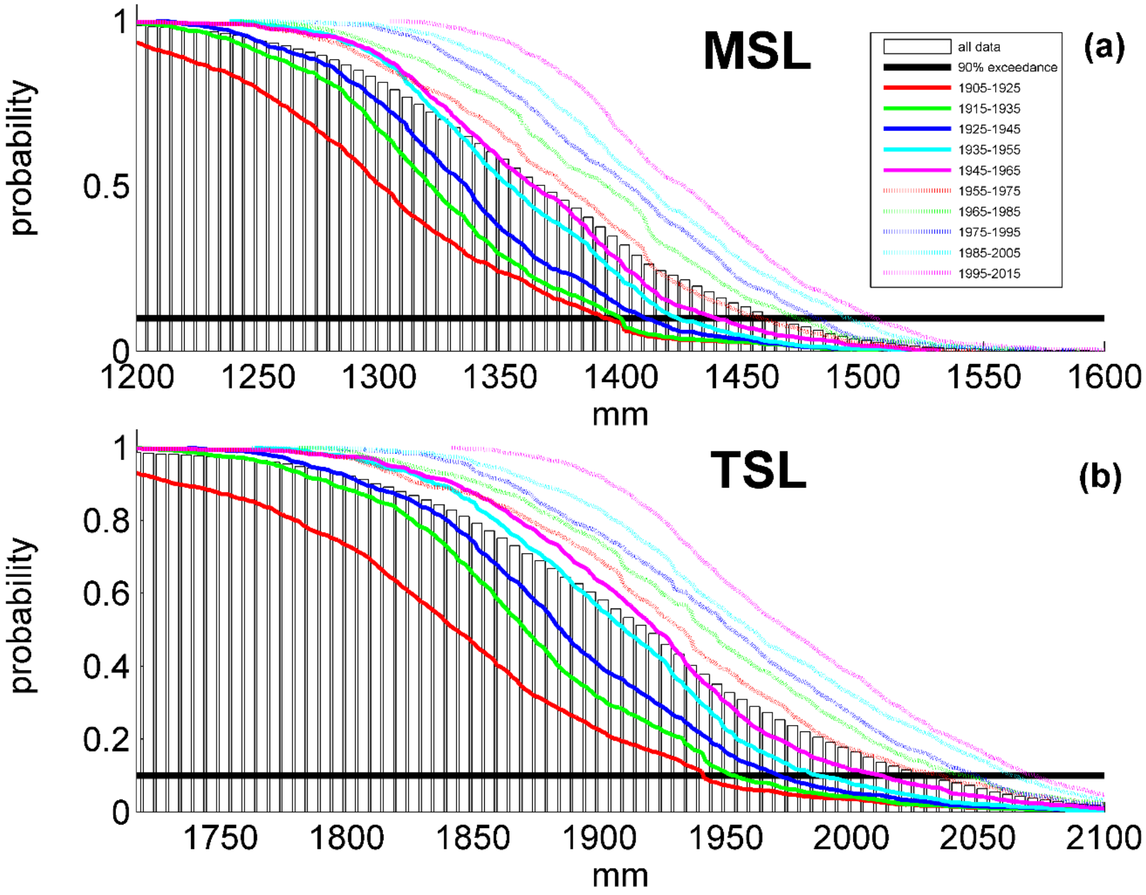

Figure S10 Probability density functions (PDFs) of: (a) mean sea level (MSL), and (b), total sea level (TSL) for the Honolulu tide gauge. In both plots, the black bar graph shows the distribution of all data, and colored lines indicate different time periods, given in the legend. The solid horizontal black line on each subplot shows the 95% exceedance level. Plots were generated using MATLAB version R2011a (www.mathworks.com).

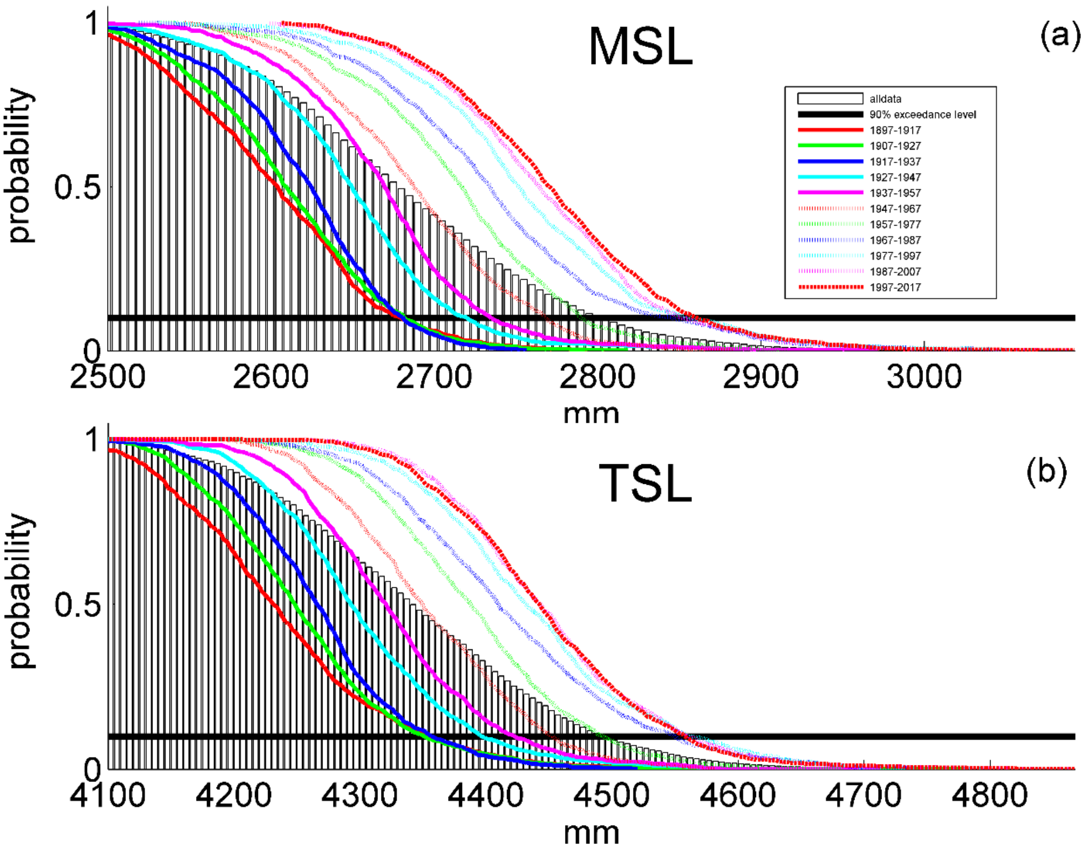

Figure S11 Probability density functions (PDFs) of: (a) mean sea level (MSL), and (b), total sea level (TSL) for the San Francisco tide gauge. In both plots, the black bar graph shows the distribution of all data, and colored lines indicate different time periods, given in the legend. The solid horizontal black line on each subplot shows the 95% exceedance level. Plots were generated using MATLAB version R2011a (www.mathworks.com).

Table S1 Station information for tidal records used in this study. Information given is: station name, latitude/longitude, country, start year of record used, end year of record used, and source agency. Locations are shown in Figure 2 and 3.[a]

**Station Name**	***Lat.(N)***	***Long. (E)***	***Country***	***Start Year***	***End Year***	***Source***
**NORTHEAST PAC**						
Fr. Frigate Shoals	23.87	-166.28	USA	1974	2014	UHI
Cabo San Lucas	22.88	-109.92	Mexico	1973	2014	UHI
Kodiak Island, AK	57.73	-152.52	USA	1975	2014	UHI
Adak, AK	51.87	-176.63	USA	1950	2014	UHI
Dutch Harbor, AK	53.88	-166.53	USA	1985	2014	UHI
Midway	28.22	-177.37	USA	1947	2014	UHI
Johnston	16.75	-169.52	USA	1947	2014	UHI
Honolulu, HI	21.3	-157.87	USA	1905	2014	UHI
Nawiliwilli Bay, HI	21.97	-159.35	USA	1954	2014	UHI
Kahului, HI	20.9	-156.47	USA	1950	2014	UHI
Hilo, HI	19.73	-155.07	USA	1927	2014	UHI
Mokuoloe, HI	21.43	-157.8	USA	1957	2014	UHI
Tofino	49.15	-125.92	Canada	1949	2014	FOC
Victoria	48.42	-123.37	Canada	1909	2014	FOC
San Francisco, CA	37.8	-122.47	USA	1899	2014	UHI
La Jolla, CA	32.87	-117.25	USA	1925	2014	UHI
Monterey, CA	36.6	-121.88	USA	1973	2014	UHI
Crescent City, CA	41.75	-124.18	USA	1933	2014	UHI
Neah Bay, WA	48.37	-124.62	USA	1934	2014	UHI
Sitka, AK	57.05	-135.35	USA	1938	2014	UHI
Seward, AK	60.12	-149.43	USA	1979	2014	UHI
Seldovia, AK	59.43	-151.72	USA	1980	2014	UHI
Valdez, AK	61.13	-146.37	USA	1973	2014	UHI
Port San Luis, CA	35.18	-120.77	USA	1983	2014	UHI
Los Angeles, CA	33.72	-118.27	USA	1924	2014	UHI
San Diego, CA	32.72	-117.17	USA	1906	2014	UHI
Yakutat, AK	59.55	-139.73	USA	1961	2014	UHI
Ketchikan, AK	55.33	-131.63	USA	1949	2014	UHI
Astoria, OR	46.22	-123.77	USA	1925	2014	UHI
Charleston, OR	43.35	-124.32	USA	1978	2014	UHI
Santa Monica, CA	34.02	-118.5	USA	1973	2014	UHI
Cordova, AK	60.57	-145.75	USA	1978	2014	UHI
South Beach, OR	44.63	-124.05	USA	1967	2014	UHI
Seattle	47.6	-122.4	USA	1899	2011	UHI
Vancouver	49.29	-123.11	Canada	1943	2014	FOC
Point Atkinson	49.34	-123.25	Canada	1960	2014	FOC

Bella Bella	52.16	-128.14	Canada	1962	2014	FOC
Queen Charlotte	53.25	-132.07	Canada	1966	2014	FOC
Port Hardy	50.72	-127.49	Canada	1965	2014	FOC
Bamfield	48.84	-125.14	Canada	1971	2014	FOC
**SOUTHEAST PAC**						
Baltra	-0.43	-90.28	Ecuador	1985	2014	UHI
Papeete (Tahiti)	-17.53	-149.57	Fr. Poly.	1969	2014	UHI
Juan Fernandez Is.	-33.62	-78.83	Chile	1988	2014	UHI
Easter Is.	-27.15	-109.45	Chile	1970	2014	UHI
Rarotonga	-21.2	-159.78	Cook Is.	1977	2014	UHI
Penrhyn	-8.98	-158.05	Cook Is.	1977	2014	UHI
Santa Cruz	-0.75	-90.32	Ecuador	1978	2014	UHI
San Felix	-26.28	-80.13	Chile	1987	2014	UHI
Nuku' alofa	-21.13	-175.17	Tonga	1990	2014	UHI
Antofagasta	-23.65	-70.4	Chile	1945	2014	UHI
Valparaiso	-33.03	-71.63	Chile	1944	2014	UHI
Lobos de Afuera	-6.93	-80.72	Peru	1982	2010	UHI
Buena Ventura	3.9	-77.1	Colombia	1953	2011	UHI
Caldera	-27.07	-70.83	Chile	1980	2014	UHI
La Libertad	-2.2	-80.92	Ecuador	1949	2014	UHI
Callao	-12.05	-77.15	Peru	1950	2014	UHI
Matarani	-17	-72.12	Peru	1992	2014	UHI
Balboa	8.97	-79.57	Panama	1907	2014	UHI
Tumaco	1.83	-78.73	Colombia	1951	2012	UHI
Puerto Montt	-41.48	-72.97	Chile	1980	2014	UHI
**NORTHWEST PAC.**						
Chichijima	27.1	142.18	Japan	1975	2014	UHI
Hong Kong	22.3	114.22	China	1962	2014	UHI
Kaohsiung	22.62	120.28	Taiwan	1980	2014	UHI
Keelung	25.15	121.75	Taiwan	1980	2014	UHI
Nakanoshima	29.83	129.85	Japan	1984	2014	UHI
Abashiri	44.02	144.28	Japan	1968	2014	UHI
Hamada	34.9	132.07	Japan	1984	2014	UHI
Toyama	36.77	137.22	Japan	1967	2014	UHI
Kushiro	42.97	144.38	Japan	1963	2014	UHI
Ofunato	39.07	141.72	Japan	1962	2014	UHI
Mera	34.92	139.83	Japan	1965	2014	UHI
Kushimoto	33.47	135.78	Japan	1961	2014	UHI
Aburatsu	31.57	131.42	Japan	1961	2014	UHI
Naha	26.22	127.67	Japan	1966	2014	UHI
Maisaka	34.68	137.62	Japan	1968	2014	UHI
Miyakejima	34.07	139.48	Japan	1964	2014	UHI

Naze	28.38	129.5	Japan	1957	2014	UHI
Wakkanai	45.4	141.68	Japan	1967	2014	UHI
Nagasaki	32.73	129.87	Japan	1985	2014	UHI
Nishinoomote	30.73	131	Japan	1965	2014	UHI
Hakodate	41.78	140.73	Japan	1964	2014	UHI
Ishigaki	24.33	124.15	Japan	1969	2014	UHI
Hachinohe	40.53	141.53	Japan	1980	2014	UHI
Hanasaki	48.28	145.58	Japan	1977	2009	JODC
Kamaishi	39.27	141.89	Japan	1956	2010	JODC
Minamizu	34.63	138.89	Japan	1965	2008	JODC
Miyako	39.63	141.97	Japan	1967	2009	JODC
Muoran	42.34	140.95	Japan	1967	2008	JODC
Nagoya	35.08	136.88	Japan	1961	2009	JODC
Omaezaki	34.6	138.23	Japan	1961	2009	JODC
Onahama	36.93	140.92	Japan	1967	2009	JODC
Owase	34.07	136.22	Japan	1967	2009	JODC
Toba	34.47	136.85	Japan	1969	2009	JODC
Tokyo	35.67	139.77	Japan	1968	2009	JODC
Urigami	33.55	135.9	Japan	1969	2009	JODC
Odomari	31.02	130.69	Japan	1965	2009	JODC
Okada	34.78	139.4	Japan	1963	2009	JODC
Shimizuminato	35.02	138.5	Japan	1965	2009	JODC
Shirihama	33.68	135.38	Japan	1968	2009	JODC
Tosashimizu	32.78	132.97	Japan	1961	2009	JODC
**SOUTHWEST PAC.**						
Pohnpei	6.98	158.25	Micronesia	1974	2014	UHI
Nauru	-0.53	166.92	Rep of Nauru	1975	2014	UHI
Majuro	7.12	171.37	Rep Marshall Is	1968	2014	UHI
Malakal	7.33	134.47	Rep of Belau	1969	2014	UHI
Yap	9.52	138.13	Fd St Micronesia	1969	2014	UHI
Honiara	-9.42	159.95	Solomon Islands	1974	2014	UHI
Rabaul	-4.2	152.18	Pap. New Guinea	1966	1997	UHI
Christmas Island	1.98	-157.47	Rep of Kiribati	1974	2014	UHI
Suva	-18.13	178.43	Fiji	1972	2014	UHI
Noumea	-22.3	166.43	France	1967	2014	UHI
Funafuti	-8.5	179.22	Fiji	1977	2014	UHI
Saipan	15.23	145.75	N. Mari. Islands	1978	2014	UHI
Kapingamarangi	1.1	154.78	Fd St Micronesia	1978	2009	UHI
Port Villa	-17.77	168.3	Vanuatu	1993	2014	UHI
Wake	19.28	166.62	USA	1950	2014	UHI
Guam	13.43	144.65	Guam	1948	2014	UHI
Kwajalein	8.73	167.73	Marshall Islands	1946	2014	UHI

Pago Pago	-14.28	-170.68	USA	1948	2014	UHI
Manus Island	-2.02	147.27	Pap. New Guinea	1993	2014	CSIRIO
Wellington	-41.28	174.78	New Zealand	1944	2014	UHI
Cendering	5.27	103.18	Malaysia	1984	2014	UHI
Johor Bahru	1.47	103.8	Malaysia	1984	2014	UHI
Kuantan	3.98	103.43	Malaysia	1984	2014	UHI
Tioman	2.8	104.13	Malaysia	1985	2014	UHI
Sedili	1.93	104.12	Malaysia	1986	2014	UHI
Kukup	1.33	103.45	Malaysia	1985	2014	UHI
Getting	6.23	102.1	Malaysia	1986	2014	UHI
Ko Lak	11.8	99.82	Thailand	1985	2014	UHI
Tanjong Pagar	1.27	103.85	Singapore	1988	2014	UHI
Kelang	3.05	101.37	Malaysia	1984	2014	UHI
Kaling	2.22	102.15	Malaysia	1984	2014	UHI
Langkawi	6.43	99.75	Malaysia	1985	2014	UHI
Lumut	4.23	100.62	Malaysia	1984	2014	UHI
Penang	5.42	100.35	Malaysia	1984	2014	UHI
Ko Taphao Noi	7.83	98.43	Thailand	1985	2014	UHI
Vung Tau	10.33	107.07	Vietnam	1986	2014	UHI
Kota Kinabalu	5.98	116.07	Malaysia	1987	2014	UHI
Bintulu	3.22	113.07	Malaysia	1992	2014	UHI
Sandakan	5.82	118.07	Malaysia	1993	2014	UHI
Brisbane	-27.37	153.17	Australia	1984	2014	UHI
Bundaberg	-24.83	152.35	Australia	1984	2014	UHI
Ft. Denison (Sydney)	-33.85	151.23	Australia	1965	2014	UHI
Townsville	-19.25	146.83	Australia	1984	2014	UHI
Spring Bay	-42.55	147.93	Australia	1985	2014	UHI
Booby Island	-10.6	141.92	Australia	1988	2014	UHI
Hobart	-42.88	147.33	Australia	1985	2012	UHI
Manila	14.58	120.97	Philippines	1984	2012	UHI
Legaspi	13.15	123.75	Philippines	1984	2014	UHI
Davao	7.08	125.63	Philippines	1984	2014	UHI
Lord Howe Is.	-31.52	159.07	Australia	1991	2014	UHI
Lautoka	-17.6	177.43	Fiji	1992	2014	UHI
Cairns	-16.92	145.77	Australia	1984	2014	AuNTC
Gladstone	-23.85	151.26	Australia	1978	2014	AuNTC
Williamstown	-37.86	144.89	Australia	1966	2014	AuNTC

[a] –Source agency abbreviations: UHI: University of Hawaii Sea level Center; FOC: Fisheries and Oceans Canada; LINZ: Land Information New Zealand; CSIRO: Commonwealth Scientific and Research Organization; AuNTC: Australian National Tidal Center.

Table S2 Pacific Ocean changes in the highest astronomical tide (δ-HAT) for a 1-meter fluctuation in MSL, based on the detrended combined tidal variability of the M_2, S_2, K_1, and O_1 tides, as compared to the detrended yearly MSL variability. δ-HAT values indicate the average of a set of yearly-windowed harmonic analyses, 12 in total (Jan-Dec, Feb-Jan, etc). Confidence intervals (CI) represent the interquartile range of the δ-HAT determination set, and the *p*-value is an indicator of significance. Significant results (*p*-value < .05, δ-HAT > CI, and magnitude > ±50 mm m^{-1}) are indicated by bold text[a].

Station Name	δ-HAT	CI (±)	*p-value*
French Frigate Sh., USA	**-52.62**	**16.89**	**0.001**
Cabo San Lucas, MEX	57.75	64.52	0.094
Kodiak Island, AK	12.27	18.80	0.124
Adak, AK	11.60	17.66	0.400
Dutch Harbor, AK	**-61.00**	**17.63**	**0.005**
Midway, USA	-15.74	15.04	0.380
Johnston, USA	**-116.88**	**24.45**	**0.001**
Honolulu, HI	**139.51**	**21.62**	**< 0.001**
Nawiliwilli Bay, HI	**55.61**	**12.27**	**0.018**
Kahului, HI	-13.41	7.19	0.059
Hilo, HI	**146.75**	**12.18**	**< 0.001**
Mokuoloe, HI	44.24	25.30	0.014
Tofino, CAN	13.39	31.77	0.163
Victoria, CAN	-60.30	43.39	0.158
San Francisco, CA	**-146.56**	**35.20**	**< 0.001**
La Jolla, CA	**59.50**	**34.60**	**0.001**
Monterey, CA	27.37	22.30	0.016
Crescent City, CA	-48.44	12.73	0.090
Neah Bay, WA	9.80	14.06	0.246
Sitka, AK	-23.03	39.63	0.107
Seward, AK	-8.19	25.83	0.287
Seldovia, AK	**-53.76**	**40.79**	**0.009**
Valdez, AK	52.06	18.79	0.184
Port San Luis, CA	4.49	18.17	0.365
Los Angeles, CA	-21.39	17.05	0.079
San Diego, CA	-17.77	17.18	0.143
Yakutat, AK	21.99	41.13	0.015
Ketchikan, AK	21.89	15.73	0.024
Astoria, OR	**-256.81**	**35.09**	**< 0.001**
Charleston, OR	-17.47	18.59	0.393
Santa Monica, CA	**-51.72**	**47.75**	**0.030**
Cordova, AK	16.24	14.86	0.512

South Beach, OR	-16.05	40.56	0.025
Seattle, WA	-34.64	37.09	0.207
Vancouver, CAN	-160.29	53.48	0.074
Point Atkinson, CAN	-97.58	232.94	0.084
Bella Bella, CAN	-35.24	33.36	0.125
Queen Charlotte, CAN	1.47	71.85	0.470
Port Hardy, CAN	-12.79	36.92	0.636
Bamfield, CAN	11.02	26.28	0.022
Baltra, ECU	26.89	18.15	0.122
Papeete (Tahiti), PYF	**-95.29**	**43.32**	**0.008**
Juan Fernandez Is., CHL	-28.68	11.47	0.283
Easter Is., CHL	28.73	24.75	0.217
Rarotonga, COK	49.97	20.53	0.227
Penrhyn, COK	31.33	18.75	0.004
Santa Cruz, ECU	-34.78	25.26	0.666
San Felix, CHL	**79.63**	**36.25**	**0.010**
Nuku' alofa, TON	**-103.31**	**10.30**	**0.001**
Antofagasta, CHL	-33.28	5.49	0.045
Valparaiso, CHL	**-122.85**	**38.33**	**0.003**
Lobos de Afuera, PER	23.92	16.59	0.690
Buena Ventura, COL	-81.61	16.93	0.113
Caldera, CHL	**127.40**	**27.28**	**0.004**
La Libertad, ECU	**-73.11**	**16.75**	< **0.001**
Matarani, PER	-40.29	22.12	0.042
Balboa, PAN	**-56.77**	**11.19**	**0.004**
Tumaco, COL	33.97	21.07	0.001
Puerto Montt, CHL	**-963.21**	**107.66**	< **0.001**
Chichijima, JPN	-17.09	20.63	0.758
Hong Kong, HKG	**665.44**	**99.23**	**0.002**
Kaohsiung, JPN	-17.48	16.91	0.771
Keelung, JPN	-54.28	53.95	0.072
Nakanoshima, JPN	47.39	36.07	0.380
Abashiri, JPN	**-126.15**	**47.40**	**0.003**
Hamada, JPN	56.95	83.61	0.074
Toyama, JPN	-29.18	37.40	0.002
Kushiro, JPN	-43.71	22.09	0.008
Ofunato, JPN	-78.73	32.98	0.052
Mera, JPN	**-100.04**	**9.57**	**0.004**
Kushimoto, JPN	-25.03	26.31	0.460
Aburatsu, JPN	36.18	12.17	0.190
Naha, JPN	39.46	18.16	0.131
Maisaka, JPN	**-350.29**	**29.25**	< **0.001**
Miyakejima, JPN	**-55.27**	**8.44**	**0.001**

Naze, JPN	-29.62	29.23	0.328
Wakkanai, JPN	**-108.48**	**22.70**	**0.015**
Nagasaki, JPN	-85.53	28.09	0.214
Nishinoomote, JPN	14.34	34.65	0.948
Hakodate, JPN	11.96	24.16	0.397
Ishigaki, JPN	**-85.53**	**17.11**	**0.005**
Hachinohe, JPN	**-82.28**	**23.08**	**0.006**
Hanasaki, JPN	**53.59**	**17.67**	**0.008**
Kamaishi, JPN	-13.60	49.69	0.503
Minamizu, JPN	-15.73	23.52	0.968
Miyako, JPN	-18.20	9.85	0.802
Nagoya, JPN	2.49	7.05	0.807
Omaezaki, JPN	-29.21	38.98	0.199
Onahama, JPN	-42.33	31.63	0.050
Owase, JPN	-3.58	12.84	0.644
Toba, JPN	-85.16	14.55	0.621
Tokyo, JPN	**-137.30**	**31.50**	**0.008**
Urigami, JPN	32.69	18.72	0.191
Odomari, JPN	**-193.72**	**52.20**	**0.001**
Okada, JPN	**158.85**	**20.41**	**0.005**
Shimizuminato, JPN	**-208.53**	**54.03**	**0.010**
Shirihama, JPN	-38.17	34.38	0.054
Tosashimizu, JPN	**-81.37**	**56.50**	**0.013**
Pohnpei, FSM	-6.22	14.96	0.861
Nauru, NRU	-53.89	7.83	0.246
Majuro, MHL	-26.84	20.39	0.356
Malakal, PLW	4.34	24.61	0.786
Yap, FSM	**-57.78**	**25.15**	**0.006**
Honiara, SLB	**66.79**	**10.13**	**< 0.001**
Rabaul, PNG	30.36	7.86	0.040
Christmas Island, KIR	**-70.51**	**8.38**	**0.004**
Suva, FJI	**71.90**	**68.46**	**0.001**
Noumea, FRA	73.12	28.04	0.558
Funafuti, TUV	-26.57	30.14	0.084
Saipan, NMP	-5.84	14.93	0.640
Kapingamarangi, FSM	15.71	15.00	0.883
Port Villa, VUT	74.19	52.38	0.197
Wake, USA	-24.16	31.65	0.759
Guam, USA	**-115.92**	**17.85**	**< 0.001**
Kwajalein, MHL	38.25	12.64	0.009
Pago Pago, USA	28.16	21.31	0.119
Manus Island, PNG	0.36	51.64	0.448
Wellington, NZL	-39.35	17.88	0.164

Cendering, MYS	**-131.72**	**67.22**	**0.025**
Johor Bahru, MYS	-232.08	36.09	0.146
Kuantan, MYS	**-216.82**	**53.51**	**0.001**
Tioman, MYS	**-292.35**	**48.19**	**0.018**
Sedili, MYS	**-215.36**	**107.81**	**0.003**
Kukup, MYS	**-184.56**	**59.35**	**0.014**
Getting, MYS	-83.72	103.62	0.370
Ko Lak, THA	-42.28	53.26	0.171
Tanjong Pagar, SGP	4.46	9.54	0.934
Kelang, MYS	**-152.29**	**34.13**	**0.010**
Kaling, MYS	**-218.47**	**57.52**	**0.004**
Langkawi, MLY	**-143.79**	**37.15**	**< 0.001**
Lumut, MLY	**-116.95**	**42.95**	**0.012**
Penang, MLY	**-138.73**	**29.69**	**0.001**
Ko Taphao Noi, THA	**-70.35**	**32.54**	**0.016**
Vung Tau, VNM	108.00	87.06	0.104
Kota Kinabalu, MYS	**-67.18**	**23.90**	**0.028**
Bintulu, MYS	**614.89**	**140.46**	**< 0.001**
Sandakan, MYS	**107.56**	**28.17**	**0.011**
Brisbane, AUS	**272.56**	**39.62**	**0.009**
Bundaberg, AUS	-13.85	19.94	0.756
Ft. Denison (Sydney), AUS	-2.46	20.72	0.650
Townsville, AUS	-11.58	12.32	0.513
Spring Bay, AUS	-87.50	80.18	0.105
Booby Island, AUS	19.60	79.56	0.786
Hobart, AUS	27.53	23.14	0.856
Manila, PHL	-118.76	66.57	0.108
Legaspi, PHL	**-144.79**	**36.48**	**0.013**
Davao, PHL	68.99	89.72	0.938
Lord Howe Is., AUS	10.24	27.38	0.246
Lautoka, FJI	**53.80**	**19.66**	**0.387**
Cairns, AUS	297.56	1274.52	0.719
Gladstone, AUS	-40.45	300.49	0.631
Williamstown, AUS	145.75	568.48	0.912

[a] -All values are expressed as millimeter change in δ-HAT per meter fluctuation in MSL (mmm^{-1}). Significant values are in bold text, based on a SNR > 2, and an absolute magnitude of > 50 mmm^{-1}. Country location is given by three-letter abbreviations, U.S. states are indicated by two-letter code.

Table S3 Exceedance levels at Hong Kong of: diurnal tides (D_1), semidiurnal tides (D_2), overtides (OT), mean sea level (MSL),, and total sea levels ($D_1 + D_2$ + OT + MSL; 'TSL'). For all determinations, the 90%, 95%, and 99% levels are given for the first part of the historical tide gauge record (1965-1985), the last part of the record (1995-2015), and the difference between the two times to show the change in each exceedance level. All values are in units of millimeters.

	1965-1985 level (mm)	***1995-2015 level (mm)***	***Change in level (mm)***
D_1 90% exceedance level	866	904	**+38**
D_1 95% exceedance level	877	913	**+36**
D_1 99% exceedance level	900	938	**+38**
D_2 90% exceedance level	775	793	**+18**
D_2 95% exceedance level	782	804	**+22**
D_2 99% exceedance level	797	820	**+23**
OT 90% exceedance level	185	232	**+47**
OT 95% exceedance level	194	245	**+51**
OT 99% exceedance level	213	272	**+59**
MSL 90% exceedance level	1521	1611	**+90**
MSL 95% exceedance level	1584	1662	**+78**
MSL 99% exceedance level	1665	1740	**+75**
TSL 90% exceedance level	3260	3430	***+170***
TSL 95% exceedance level	3330	3480	***+150***
TSL 99% exceedance level	3435	3557	***+122***

Table S4 Exceedance levels at Honolulu of: diurnal tides (D_1), semidiurnal tides (D_2), overtides (OT), mean sea level (MSL), and total sea levels ($D_1 + D_2$ + OT + MSL; 'TSL'). For all determinations, the 90%, 95%, and 99% levels are given for the first part of the historical tide gauge record (1905-1925), the last part of the record (1995-2015), and the difference between the two times to show the change in each exceedance level. All values are in units of millimeters.

	1905-1925 level (mm)	***1995-2015 level (mm)***	***Change in level (mm)***
D_1 90% exceedance level	290	293	**+3**
D_1 95% exceedance level	299	301	**+2**
D_1 99% exceedance level	323	326	**+3**
D_2 90% exceedance level	269	281	**+12**
D_2 95% exceedance level	272	289	**+17**
D_2 99% exceedance level	3289	303	**+14**
OT 90% exceedance level	25	22	**-3**
OT 95% exceedance level	27	24	**-3**
OT 99% exceedance level	34	29	**-5**
MSL 90% exceedance level	1393	1503	**+110**
MSL 95% exceedance level	1408	1520	**+112**
MSL 99% exceedance level	1481	1564	**+83**
TSL 90% exceedance level	1941	2073	***+132***
TSL 95% exceedance level	1974	2098	***+124***
TSL 99% exceedance level	2060	2152	***+92***

Table S5 Exceedance levels at San Francisco of: diurnal tides (D_1), semidiurnal tides (D_2), overtides (OT), mean sea level (MSL), and total sea levels (D_1 + D_2 + OT + MSL; 'TSL'). For all determinations, the 90%, 95%, and 99% levels are given for the first part of the historical tide gauge record (1897-1917), the last part of the record (1995-2015), and the difference between the two times to show the change in each exceedance level. All values are in units of millimeters.

	1897-1917 level (mm)	***1995-2015 level (mm)***	***Change in level (mm)***
D_1 90% exceedance level	763	765	**+2**
D_1 95% exceedance level	794	784	**-10**
D_1 99% exceedance level	879	849	**-30**
D_2 90% exceedance level	855	901	**+46**
D_2 95% exceedance level	874	914	**+30**
D_2 99% exceedance level	921	944	**+23**
OT 90% exceedance level	139	111	**-28**
OT 95% exceedance level	151	124	**-27**
OT 99% exceedance level	168	152	**-16**
MSL 90% exceedance level	2678	2860	**+182**
MSL 95% exceedance level	2706	2894	**+188**
MSL 99% exceedance level	2776	2970	**+214**
TSL 90% exceedance level	4355	4557	***+202***
TSL 95% exceedance level	4396	4590	***+194***
TSL 99% exceedance level	4481	4708	***+227***
